# Penton-Dodecahedral Particles Trigger Opening of Intercellular Junctions and Facilitate Viral Spread during Adenovirus Serotype 3 Infection of Epithelial Cells

**DOI:** 10.1371/journal.ppat.1003718

**Published:** 2013-10-31

**Authors:** Zhuo-Zhuang Lu, Hongjie Wang, YiYi Zhang, Hua Cao, Zongyi Li, Pascal Fender, André Lieber

**Affiliations:** 1 University of Washington, Division of Medical Genetics, Seattle, Washington, United States of America; 2 Unit of Virus Host Cell Interactions, UMI3265, CNRS/EMBL/UJF, Grenoble, France; 3 University of Washington, Department of Pathology, Seattle, Washington, United States of America; University of Michigan, United States of America

## Abstract

Human adenovirus serotypes Ad3, Ad7, Ad11, and Ad14 use the epithelial junction protein desmoglein 2 (DSG2) as a receptor for infection. During Ad infection, the fiber and penton base capsid proteins are produced in vast excess and form hetero-oligomers, called pentons. It has been shown for Ad3 that pentons self-assemble into penton-dodecahedra (PtDd). Our previous studies with recombinant purified Ad3 PtDd (produced in insect cells) showed that PtDd bind to DSG2 and trigger intracellular signaling resulting in the transient opening of junctions between epithelial cells. So far, a definitive proof for a function of Ad3 PtDd in the viral life cycle is elusive. Based on the recently published 3D structure of recombinant Ad3 PtDd, we generated a penton base mutant Ad3 vector (mu-Ad3GFP). mu-Ad3GFP is identical to its wild-type counterpart (wt-Ad3GFP) in the efficiency of progeny virus production; however, it is disabled in the production of PtDd. For infection studies we used polarized epithelial cancer cells or cell spheroids. We showed that in wt-Ad3GFP infected cultures, PtDd were released from cells before viral cytolysis and triggered the restructuring of epithelial junctions. This in turn facilitated lateral viral spread of *de novo* produced virions. These events were nearly absent in mu-Ad3GFP infected cultures. Our *in vitro* findings were consolidated in mice carrying xenograft tumors derived from human epithelial cancer cells. Furthermore, we provide first evidence that PtDd are also formed by another DSG2-interacting Ad serotype, the newly emerged, highly pathogenic Ad14 strain (Ad14p1). The central finding of this study is that a subgroup of Ads has evolved to generate PtDd as a strategy to achieve penetration into and dissemination in epithelial tissues. Our findings are relevant for basic and applied virology, specifically for cancer virotherapy.

## Author Summary

We have recently reported that a group of human Ads uses DSG2 as a receptor for infection. Among the DSG2-interacting Ads is serotype 3, which is widely distributed in the human population. During Ad3 infection, subviral particles (PtDd) formed by two capsid proteins are produced in vast excess and released early in infection. In this study, we demonstrate that PtDd trigger the opening of epithelial junctions and thus support the lateral spread of Ad3 progeny virus in epithelial tissues. Our study contributes to a better understanding of Ad3 infection and pathology. It also has implications for Ad-mediated gene transfer into epithelial tissues and tumors.

## Introduction

The main structural proteins of the icosahedral capsids of adenoviruses (Ads) are the hexon and penton base. The penton base forms pentamers located at the 12 vertices of the Ad particle. Each pentamer anchors one copy of a trimeric fiber protein. The C-terminal part of the fibers, the fiber knob, mediates the high affinity binding to a cellular receptor, while the RGD containing loops within the penton base interact with cellular integrins, a step that mediates cell entry of virions, except species B Ads. Most human Ad serotypes use CAR as a primary attachment receptor. Species B Ad serotypes use either CD46 or DSG2. Among DSG2-targeting viruses is serotype Ad3. Recently, we have shown that complete inhibition of Ad3 binding and infection requires the physical linkage and, most likely, a specific spatial constellation of at least two fiber knobs [1]. This specific mode of Ad3-fiber knob-DSG2 interaction is functionally relevant for opening of junctions between epithelial cells [1], [2]. Binding of Ad3 to DSG2 triggers the autocatalytic cleavage of DSG2 and activation of pathways that are reminiscent of an epithelial-to-mesenchymal transition (EMT), including the phosphorylation of MAP kinases and the downregulation of junction proteins [2], [3], [4]. The ability to open epithelial junctions appears to be important for Ad3 penetration into and spread within epithelial tissues [1], [2], [3].

During Ad infection, the penton base and fiber proteins are produced in excess and assemble in the cytosol to form fiber-penton base hetero-oligomers called pentons [5], [6]. In the case of Ad3, twelve pentons self-assemble into dodecamers with a diameter of ∼30 nm [7]. Penton-dodecahedra (PtDd) also form in insect cells during overexpression of Ad3 penton base and fiber [8]. Western blot analysis did not indicate differences in post-translational modification of PtDd produced from baculovirus vectors in insect cells and PtDd produced from Ad3 in infected HeLa cells (**Figure S1**). The crystal structure of recombinant penton base dodecahedra has recently been delineated at 3.8 Å resolution, which allowed for the elucidation of the mechanisms of Ad3 PtDd formation [9]. PtDd self-assembly is initiated through relative weak salt bridges involving residues D100 and R425. Subsequently, an N-terminal strand exchange occurs between neighboring pentons that leads to a stable PtDd particle. Notably, strand-swapping can occur only in the context of PtDd and not in the context of the viral capsid where individual penton pentamers are separated by hexons.

During Ad3 replication, PtDd are formed at an excess of 5.5×10^6^ PtDd per infectious virus [7]. The massive production of PtDd strongly suggests that they have a role in virus infection. Notably, the main natural target for Ad3 infection is the airway epithelium. Characteristic features of airway epithelial cells are an apical-basal polarization of their cell membranes and cytoskeleton as well as tight and adherens junctions that seal the paracellular space between adjacent cells and thereby provide a barrier to pathogens.

Several lines of research indicate that PtDd facilitate the lateral spread of *de novo* produced Ad3 virions in epithelial cells. *i)* During Ad3 infection PtDd are released from infected cells, prior to the release of progeny virus, and bind to neighboring cells via DSG2 [10]. *ii)* Incubation of epithelial cells with recombinant PtDd produced in insect cells results in clustering of DSG2, which in turn triggers signaling pathways that are reminicent of EMT and leads to transient opening of tight junctions between epithelial cells [2]. *iii)* Efficient binding to DSG2 and triggering of junction opening requires multiple Ad3 fiber knobs in a specific spatial constellation, which is present in PtDd, implying that dodecamerization is functionally important [1]. While these studies performed with recombinant PtDd are indicative, they do not prove a role of PtDd produced *de novo* during Ad3 infection of epithelial cells. To provide such proof we generated an Ad3 virus that was greatly disabled in the formation of PtDd and tested its effect on viral spread in epithelial cells *in vitro* and *in vivo* in comparison to a wild-type Ad3 virus.

## Results

**Figure 1:**
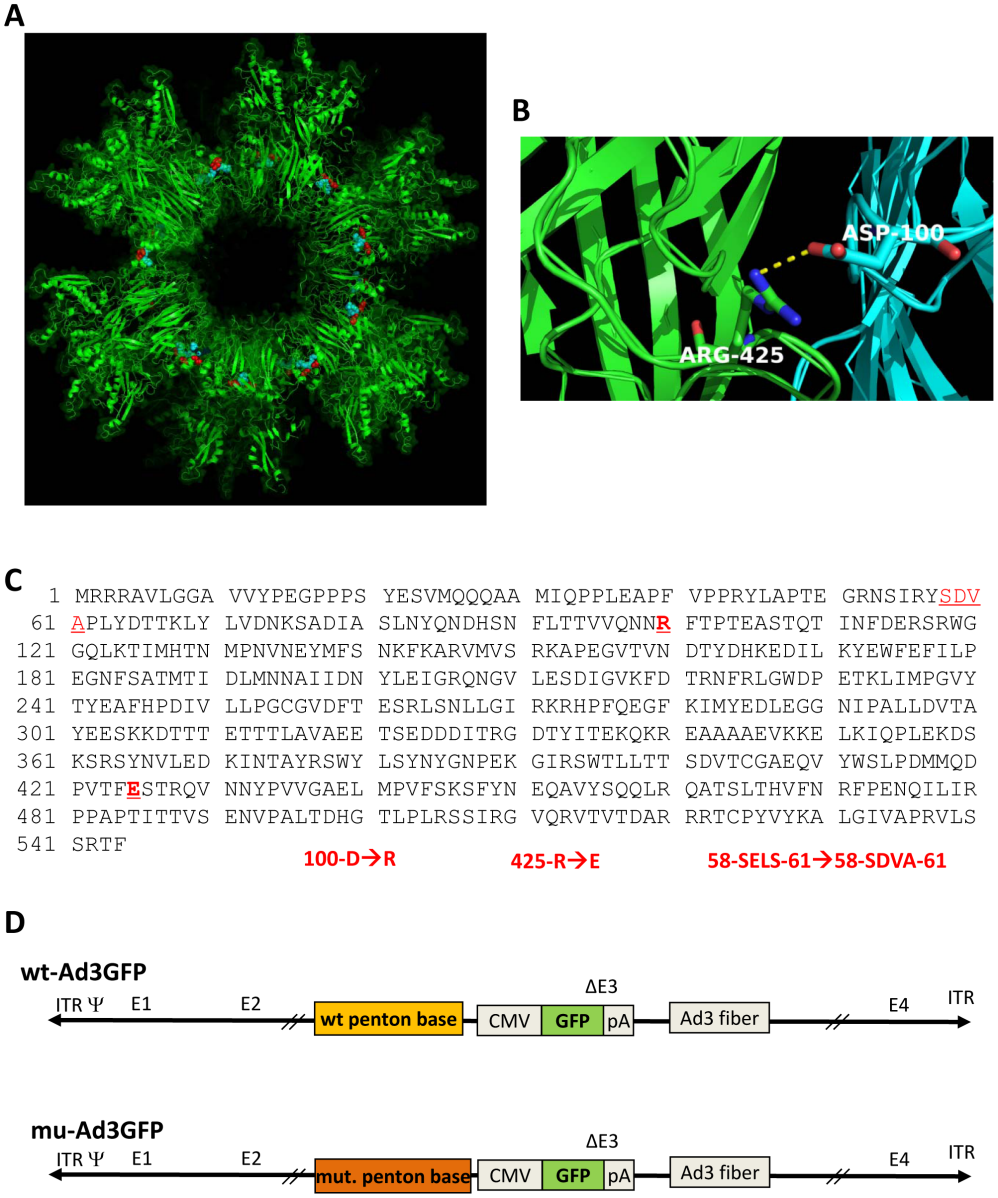
Ad3 penton base mutations that prevent the formation of PtDd. **A**) Localization of D100 (in red) and R425 (in blue) in the Ad3 dodecahedron context (PDB accession 4AQQ). The panel shows a representative view of contacts taking place at the interface between adjacent pentamers in a slab view. **B**) Two out the five monomers constitutive of a penton base are coloured in blue and green with D100 and R425 highlighted. The salt bridge between these monomers is shown. **C**) Amino acids sequence of Ad3 penton base. Substitutions of 58-SELS-61→58-SDVA-61, 100-D→R and 425-R→E were introduced into wild-type Ad3 penton base. The mutated residues are shown in red letters. **D**) Ad3 vectors. Both vectors contain the same GFP expression cassette inserted into the E3 region of Ad3 in front of the fiber sequence. Both vectors contain the complete E1 regions and are therefore replication-competent. mu-Ad3GFP contains both 100-D→R and 425-R→E mutations in the penton base.

**Figure 2:**
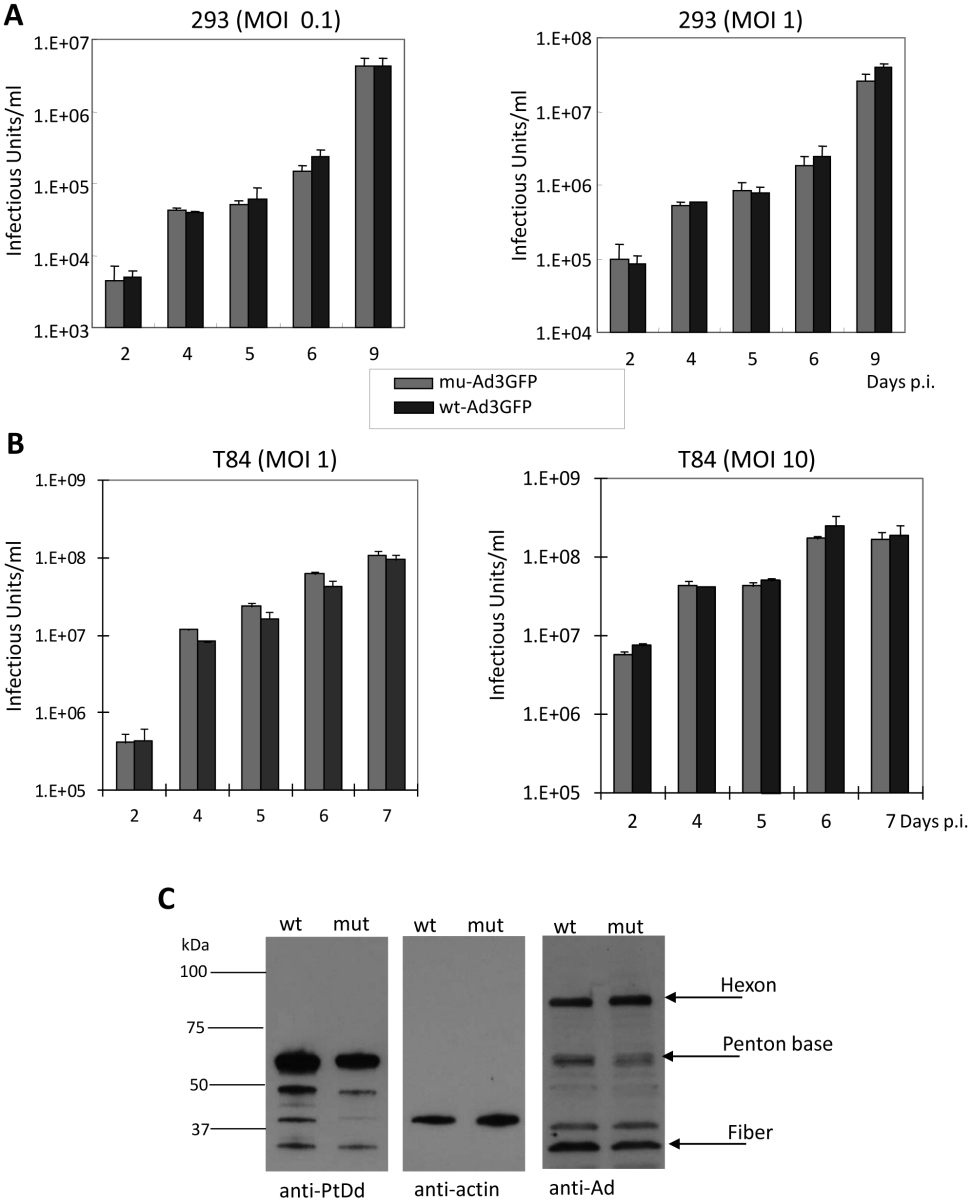
Production of wt-Ad3GFP and mu-Ad3GFP. **A**) 293 cells were infected at indicated MOIs (vp/cell) for 2 hours. Medium samples were collected at indicated time points. Viruses were titrated on 293 cells based on GFP-expressing units (“Infectious Units”). N = 3. **B**) Subconfluent T84 cells were infected and progeny virus production was measured as described in A). **C**) Comparison of penton, fiber, and hexon expression. HeLa cells infected with wt-Ad3GFP or mu-Ad3GFP were collected at 36 hours post-infection and total cell lysates (without prior ultracentrifugation) were subjected to Western blot with anti-PtDd antibodies (recognize Ad3 penton base and fiber), anti-Ad antibodies (recognize Ad3 hexon, penton base, and fiber) and anti-actin (loading control) antibodies.

A previous study using recombinant penton base dodecahedra showed that the deletion of the first 60 amino acids or the substitution of amino acid residues 58-SELS-61 to 58-SDVA-61 prevented dodecamerization. Furthermore, penton base mutants 100-D→R or 425-R→E drastically reduced the yield and stability of penton base dodecamers [9]. We therefore attempted to generate Ad3 penton base mutants that contained the SELS→SDVA mutation, the 100-D→R mutation, and the 425-R→E mutation individually or in combination (**Figures 1A–C**). The mutations were introduced into a vectorized Ad3 genome, where the parental Ad3 vector (wt-Ad3GFP) contained a CMV promoter-driven GFP gene inserted into the E3 region [2]. Mutations were introduced into the penton base sequence of wt-Ad3GFP. The correctness of the penton base gene modifications was confirmed by PCR and DNA sequencing (**Figure S2**). The recombinant viral genomes were transfected into 293 cells for virus rescue. Single plaques were then amplified and viral genomes sequenced. We could only rescue viruses that contained the D100R and R425E mutations individually or in combination. In our further studies, we used the Ad3 mutant that contained both the D100R and R425E mutations (mu-Ad3GFP) (**Figure 1D**). Progeny virus yields during the virus amplification were comparable between wt-Ad3GFP and mu-Ad3GFP (**Figure S3**). To ensure that the introduced mutations did not affect viral entry, DNA replication, assembly, and release of progeny virus, we performed virus growth curve assays in 293 cells (**Figure 2A**) and T84 cells that were cultured at relative low cell density to avoid the formation of intercellular junctions (**Figure 2B**). These studies showed that the kinetics and yields of progeny virus production did not significantly differ for wt-Ad3GFP and mu-Ad3GFP. This is further supported by the analysis of protein levels of Ad3 penton base, fiber, and hexon in infected cells by Western blot, which did not show differences between wt-Ad3GFP and mu-Ad3GFP at 36 hours after infection (**Figure 2C**).

**Figure 3:**
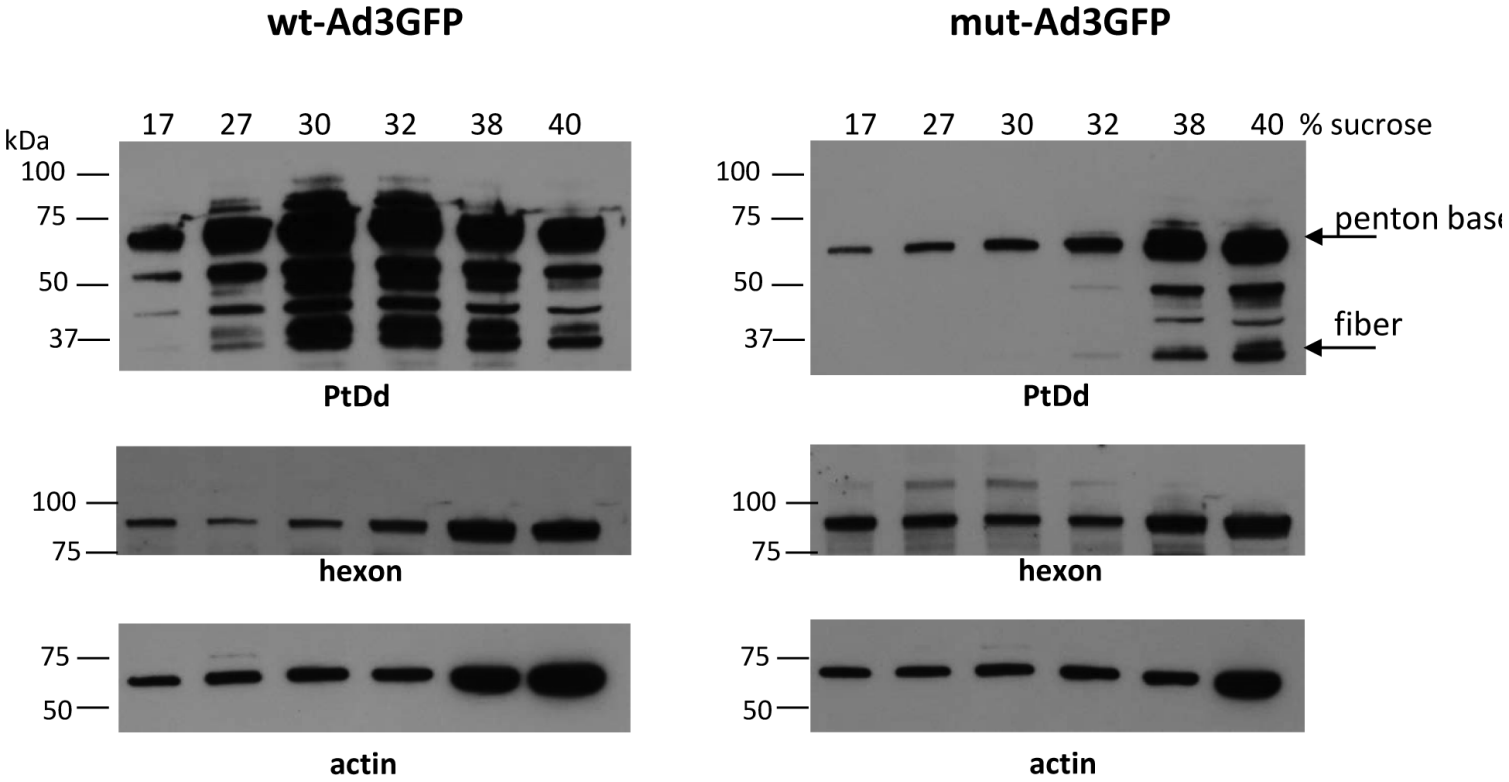
Detection of PtDd in infected cells. HeLa cells were infected with wt-Ad3GFP or mu-Ad3GFP at an MOI of 500 VP/cell. Thirty six hours later cells were collected and cell lysates subjected to ultracentrifugation in a 15–40% sucrose step gradient. Fractions with different densities were then analyzed by Western blot using polyclonal antibodies raised against purified PtDd. The antibody recognizes Ad3 penton base (61.8 kDa) and fiber (34.8 kDa). The band at ∼50 kDa is most likely a fiber derivative. Blots were also hybridized with anti-Ad antibodies that recognize Ad3 hexon (∼90 kDa) and with anti-actin antibodies (loading control).

To optimize conditions to measure PtDd production, HeLa cells were infected with wt-Ad3GFP at MOIs ranging from 500 to 2000 vp/cell. At 24, 36, and 48 hours post-infection, cell lysates were subjected to ultracentrifugation in a 15–40% sucrose step gradient. Fractions with different densities were then analyzed by Western blot using polyclonal antibodies raised against purified PtDd that reacted with both Ad3 penton base and fiber (**Figures S4 and S5**). Trace amounts of free viral penton base that was not incorporated into PtDd was found in all fractions, while PtDd concentrated in the range of 27–32% sucrose, and complete viral particles were found at the bottom of the tube in fractions 38 and 40% sucrose. The relative amount of hexon-containing defective viral particles in high density sucrose fractions was higher at 48 hours than at 24 hours and also increased with increasing MOIs. For further analysis of PtDd, we infected cells at 500 vp/cell and collected cells at 36 hours to minimize the contamination of PtDd with defective viral particles (**Figure 3**). A direct comparison of lysates from mu-Ad3GFP and wt-Ad3GFP infected cells showed markedly weaker penton base and fiber signals in 27–32% sucrose fractions for mu-Ad3GFP. Using purified viral particles and recombinant PtDd as references, we determined that the normalized PtDd signals for penton base and fibers were tenfold less intense in cell lysated from mu-Ad3GFP infected cells compared to wt-Ad3GFP infected cells (**Figure S4C**). We therefore concluded that PtDd production is severly inhibited for mu-Ad3GFP.

Since the natural target of Ad3 is polarized epithelial tissue, we then studied the spread of wt- and mu-Ad3GFP in epithelial cells. To model this, we cultured epithelial cancer cell lines under conditions that would allow for cell polarization. Colon cancer T84 cells were cultured in transwell chambers for 2–3 weeks until the trans-epithelial electrical resistance (TEER) between the inner and outer chambers was constant, i.e. mature intercellular junctions had formed. At this time, cells were exposed from the apical side with material contained in 30% sucrose fractions from wt-Ad3GFP infected cells. This lead to a decrease of the TEER within 1.5 hours after addition which indicates junction opening (**Figure S6A**). In contrast, the corresponding fraction from mu-Ad3GFP-infected cells had no significant effect on the TEER. The decrease in TEER was absent when the 30% sucrose fraction material was mixed with recombinant soluble DSG2. This suggests that the material in the fraction bound to DSG2 and therefore most likely represented PtDd (**Figure S6B**). Notably, our previous studies showed that recombinant Ad3 fiber knob (without a dimerization domain) did not significanlty affect the TEER, making it unlikely that TEER increase by the 30% sucrose fraction is caused by soluble Ad3 fiber.

**Figure 4:**
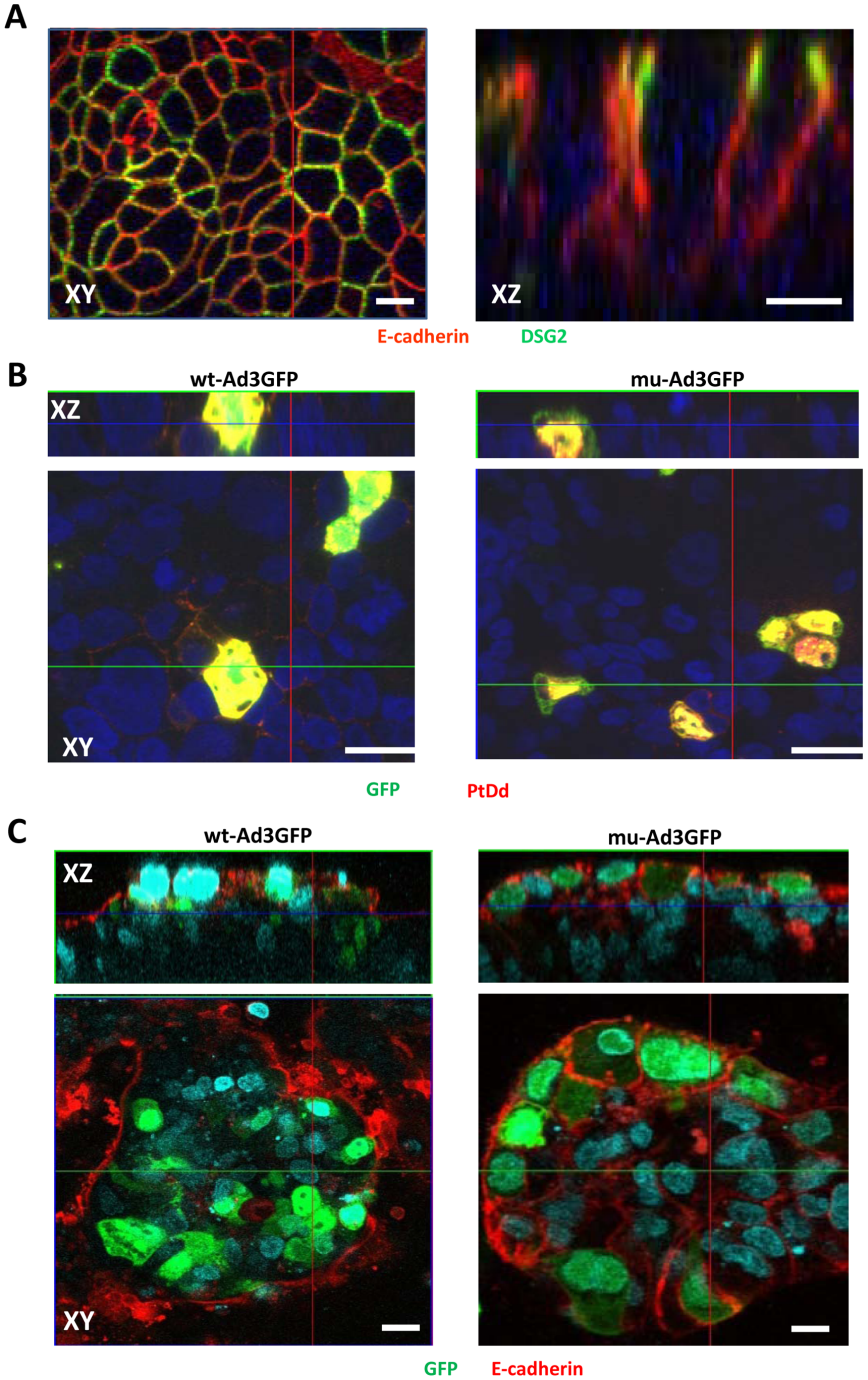
PtDd release from infected cells and effect on epithelial junctions. T84 cells were cultured in transwell chambers until the TEER was constant, i.e. epithelial junctions had formed. **A**) Confocal immunofluorescence microscopy of T84 cells. The left panel shows an XY image of the cell surface. The right panel shows stacked XZ images. Antibodies against E-cadherin (red) and DSG2 (green) visualize epithelial junction proteins. **B**) Release of PtDd from mu-Ad3GFP or wt-Ad3GFP-infected T84 cells. Polarized T84 cells were infected with mu-Ad3GFP or wt-Ad3GFP at an MOI of 10 vp/cell (added into the inner chamber) for 2 hours. 24 hours post infection, cells were fixed in 4% PFA for 15 minutes and then permeabilized with cold methanol for 10 minutes. Immunofluorescence staining was performed with anti-Ad3 PtDd polyclonal antibody (red). Nuclei were counterstained with DAPI (blue). **C**) Cells were infected as described for B). Cells were stained for the junction protein E-cadherin (red) 72 hours pos-tinfection. Virally infected cells express GFP (green). The scale bar of all images is 20 µm.

Confocal immunofluorescence microscopy analysis of T84 cells for the epithelial junction marker E-cadherin and the desmosomal protein DSG2 shows the “chicken-wire” staining of cell membranes that is typical for epithelial cells (**Figure 4A** **, left panel**), with the paracellular space sealed by junction proteins (**Figure 4A** **, right panel**). Most of the DSG2 molecules are trapped in lateral junctions and only rare T84 cells display DSG2 on the apical cell surface. This implies that only few T84 cells can be infected if Ad3 vectors are applied to the apical surface, Ad infection can be visualized based on GFP expression. GFP-positive cells co-stained with Ad3 PtDd (i.e. penton/fiber)-specific antibodies (**Figure 4B** **, red signals**) resulting in yellow signals. The yellow staining pattern suggests the *de novo* production of Ad3 virions. For wt-Ad3GFP-infected cells, PtDd-specific (red) signals can be seen in the junctions of surrounding cells (**Figure 4B** **, left panel**). This indicates release of PtDd before virus-mediated cytolysis. The latter is also supported by studies on wt-Ad3 infected HeLa cells, which do not form epithelial junctions (**Figure S7**). PtDd-specific signals were detectable in infected (GFP-postive) cells as well as in neighboring non-infected cells, indicating uptake of released PtDd by neighboring cells. Importantly, PtDd signals are visibly less pronounced outside cells in mu-Ad3GFP infected T84 cell cultures (**Figure 4B** **, right panel**). Furthermore, while junctions (visualized with anti-E-cadherin antibodies) are mostly absent in and around wt-AdGFP-infected (GFP-positive) cells, these junctions are preserved between mu-Ad3GFP infected cells (**Figure 4C**). Image morphometry for E-cadherin signals in the center of transwell cultures showed that the area of E-cadherin was 3.4(+/−2.3)% and 17.3+/−5.8% (based on mm^2^ E-cadherin per mm^2^) for wt-Ad3GFP- and mu-Ad3GFP injected mice, respectively.

Taken together, our data imply that PtDd production and subsequent junction opening is impaired in mu-Ad3GFP infected cells.

**Figure 5:**
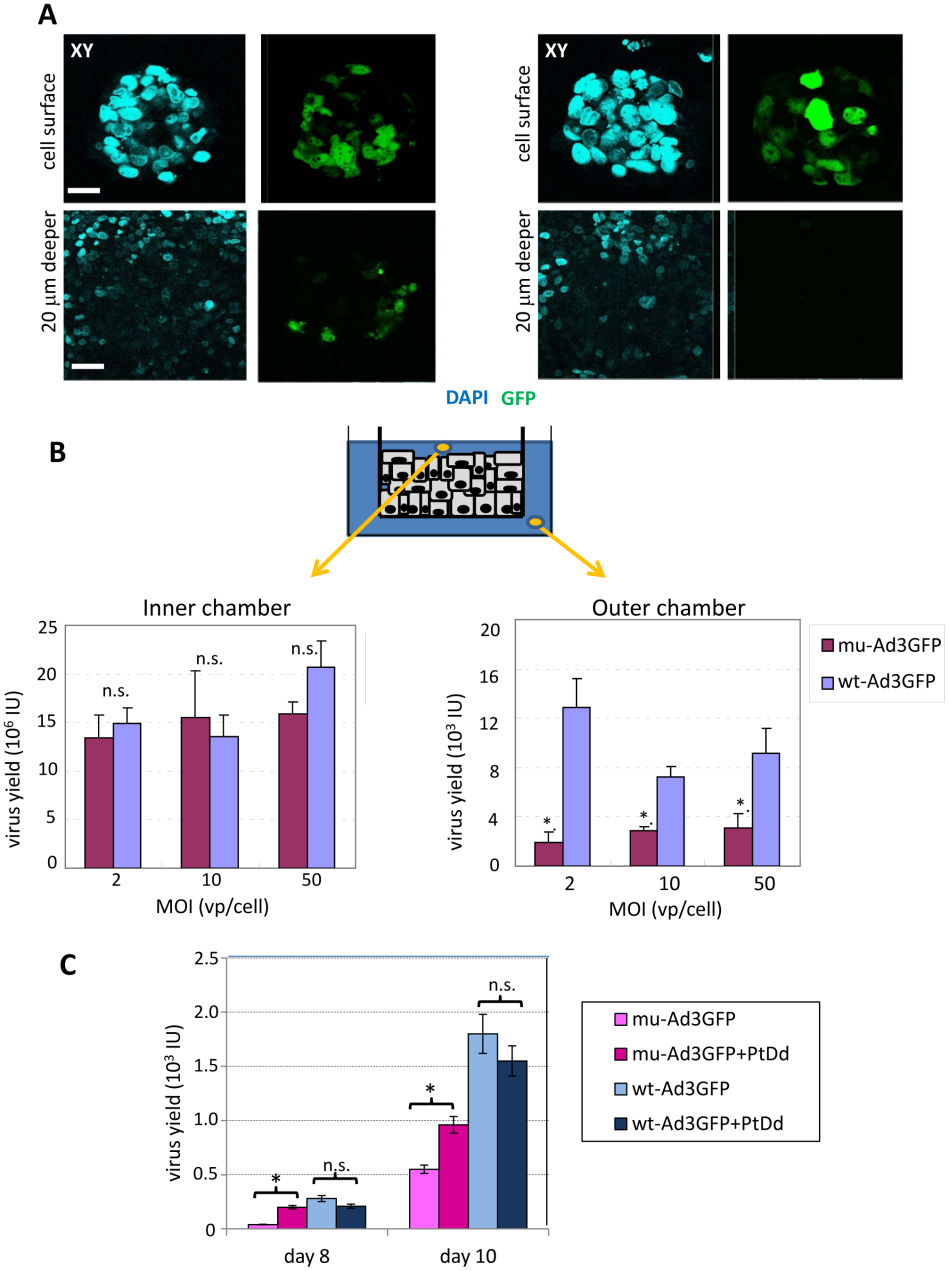
Viral spread in polarized T84 cells. **A and B**) Viral spread in T84 cells cultured in transwell chambers. Cells were cultured as described for Fig. 4. Ad vectors were added to the inner chamber at an MOI of 10 vp/cell. **A**) Confocal microscopy images (XY planes) of the cell surface and of a layer that is 20 µm beneath the cell surface were taken 4 days after infection. Transduced cells express GFP (green). The scale bar of all images is 20 µm. **B**) Titration of progeny viruses in the inner chamber and in the outer chamber. T84 cells, which had been cultured in 0.4 µm transwell inserts for 16 days, were infected from the apical side with Ad3 vectors at the indicated MOIs for 2 hours. Supernatants were collected from the inner chambers at days 4, 7, 9 and 10 post-infection, and from the outer chambers at days 9 and 10, and titered for GFP-expressing units on 293 cells. The medium were changed at days 4, 7, 9 and 10 post-infection. Shown are data from samples collected at day 9 post-infection. N = 3. The differences between wt-Ad3GFP and mu-Ad3GFP titers in the inner chamber are not significant (n.s.). *P<0.01. Virus titers in inner chamber were about 5 higher on day 7 than on day 4. Viral titers in the inner chamber remained stable from day 7 on (data not shown). Note that, the filter pore size of the chambers is 400 nm and should allow for the passage of 100 nm Ad virions). **C**) Viral spread in the presence of recombinant PtDd added to the inner chamber. T84 cells were cultured as desrcibed in B) and infected (in triplicate) with mu-Ad3GFP and wt-Ad3GFP at an MOI of 1vp/cell. Recombinant PtDd (5 µg/ml) was added to to the inner chamber on days 3, 5, and 7 post-infection. Medium in both chambers was changed on days 4, 6, 8, and 10 p.i. Day 8 and 10 medium samples from the outer chamber were then titered on 293 cells for progeny virus that has passed through the layer of T84 cells based on GFP epressing/infectious units. N = 6, *- p<0.01, n.s.-not significant.

Transwell cultures of polarized T84 cells form layers with an average thickness of five cells. Addition of wt-Ad3GFP and mu-Ad3GFP vectors resulted in the infection of the top cell layer with comparable efficiency. Confocal microscopy performed 5 days after infection revealed transduction of cells in deeper cell layers for wt-AdGFP but not for mu-Ad3GFP (**Figure 5A**). This suggests that wt-Ad3GFP produced *de novo* in primarily infected cells was able to penetrate deeper into the cell layer. Over time, progeny virus should completely spread through the multi-cell layer and be detectable at the basal side, i.e. the outer chamber of the transwell cultures. We therefore measured the titer of infectious units in the inner and outer chamber at different time points. Virus became detectable in the outer chamber only at day 9 after infection (**Figure 5B**). In the outer chamber, the titers of mu-Ad3GFP were significantly lower than those of wt-Ad3GFP. In contrast, the titers of both viruses in the inner chamber were comparable, indicating that release of *de novo* produced virus from initially infected cells in the top cell layer is not impaired for mu-Ad3GFP. The process of transepithelial spread was similar at MOI 2 and MOI 50, indicating that the cells with accessible DSG2 on the apical side were already saturated with virus at MOI 2. To further prove a role of PtDd in viral spread we added recombinant PtDd to the inner chamber of T84 cells at days 3, 5, and 7 after infection (**Figure 5C**). As in the previous experiment, less mu-Ad3GFP progeny virus was found in the outer chamber at day 8 and 10 after infection indicating that mu-Ad3GFP is impaired in viral spread. Importantly, while “exogenous” recombinant PtDd had no significant effect on the spread of wt-Ad3GFP through the layer of T84 cells, spread of mu-Ad3GFP was significantly increased. This indicates that recombinant PtDd, to some degree, compensates for the reduced PtDd production from mu-Ad3GFP.

**Figure 6:**
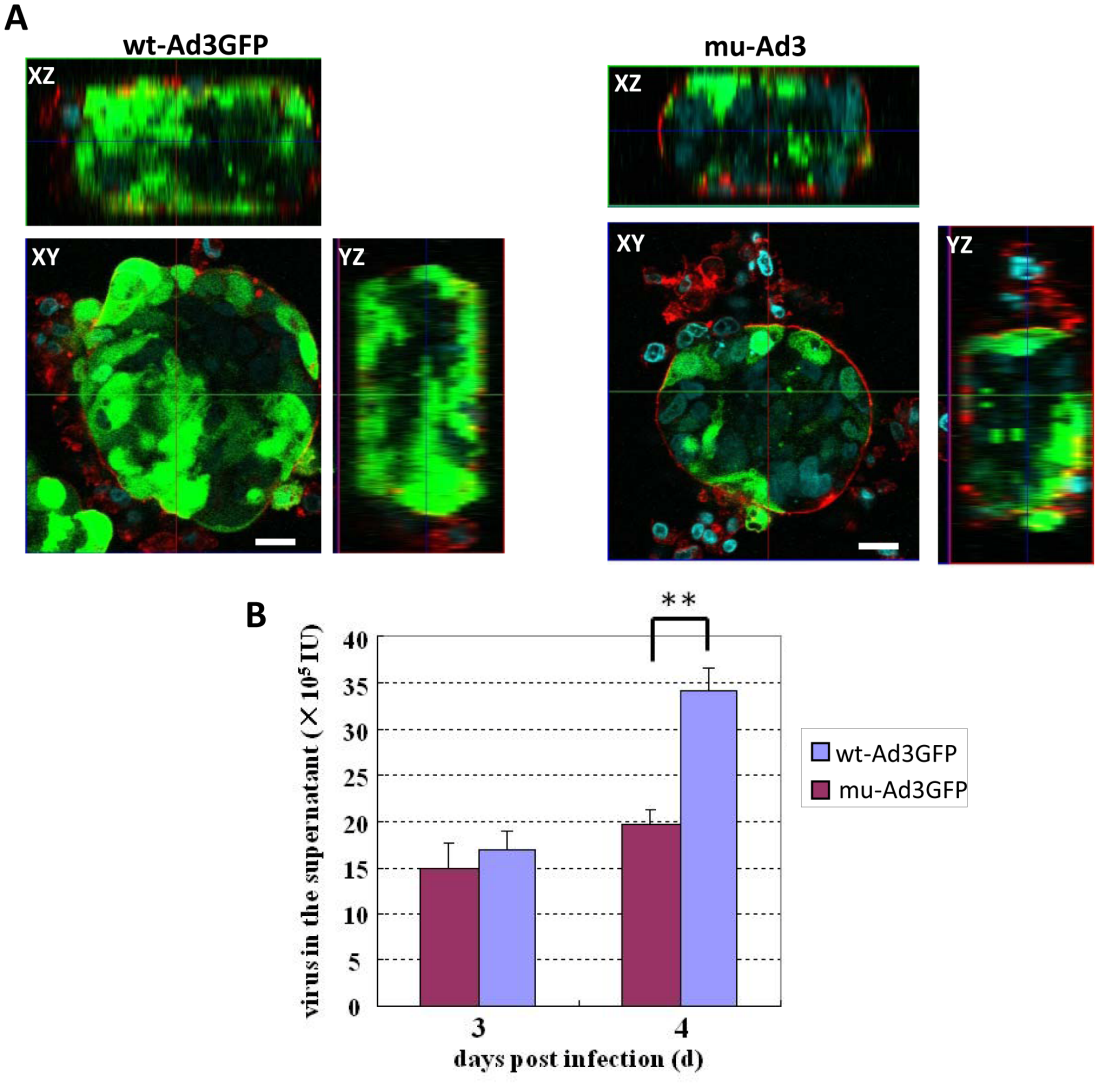
Spread of viruses in T84 cell spheroids. T84 spheroids were infected with mu-Ad3GFP or wt-Ad3GFP at an MOI of 100 vp/cell for 2 hours. Viruses were removed by washing. **A**) Spheroids were fixed with 4% PFA at day 4 post-infection. The periphery of spheroids was visualized with rhodamine-labeled Concanavalin A (Con A, red), and cellular nuclei were counterstained with DAPI (blue). Shown are XY and XZ sections. Transduced cells are GFP positive. The scale bars are 50 µm. **B**) Culture medium aliquots of the virus-infected T84 spheroids were collected at days 3 and 4 post-infection and titrated on 293 cells. N = 3 **p<0.01.

As a second model for studying viral spread, we used tumor cell spheroids that form when T84 cells are cultured in a spinner flask, which prevents their attachment to the glass surface. Culturing the cells under these conditions resulted in spheroids with ∼100 cells linked together through junctions as indicated by E-cadherin staining (**Figure S8**). wt- and mu-Ad3GFP were added to the culture for 2 hours and spheroids were analyzed by confocal microspcopy 4 days later. Transduction of peripheral cells was comparable for both viruses, while transduction of deeper cell layers was impaired for mu-Ad3GFP (**Figure 6A** **, Figure S9**). More efficient spread to, and the subsequent replication in neighboring cells should result in higher yields of do novo produced viruses in polarized T84 spheroids. We therefore measured the viral titer in the culture medium from infected T84 cell spheroids. For both viruses, titers were comparable at day 3 post-infection. Analysis at a later time point, when replication was detectable in cells based on hexon expression (data not shown), showed significantly more progeny virus was found in the culture medium of wt-Ad3GFP infected spheroids compared to mu-Ad3GFP (**Figure 6B**). More efficient dissemination of wt-Ad3GFP was also demonstrated in a second cell culture model. Human lung cancer A549 cells polarize in culture to a certain extent but do not form mature tight junctions. Viral growth kinetics in A549 cells was comparable for wt-Ad3GFP and mu-Ad3-GFP (**Figure S10A**). There was visibly less release of PtDd from mu-Ad3GFP infected cells (**Figure S10B**). wt- and mu-Ad3GFP infection of confluent A549 cells at an MOI of 0.01 vp/cell resulted in the formation of GFP expressing foci that increased over time due to viral spread. The number of foci with a diameter larger than 2 mm was significantly greater in wt-Ad3GFP infected cultures compared to mu-Ad3GFP (**Figures S10C and S10D**).

**Figure 7:**
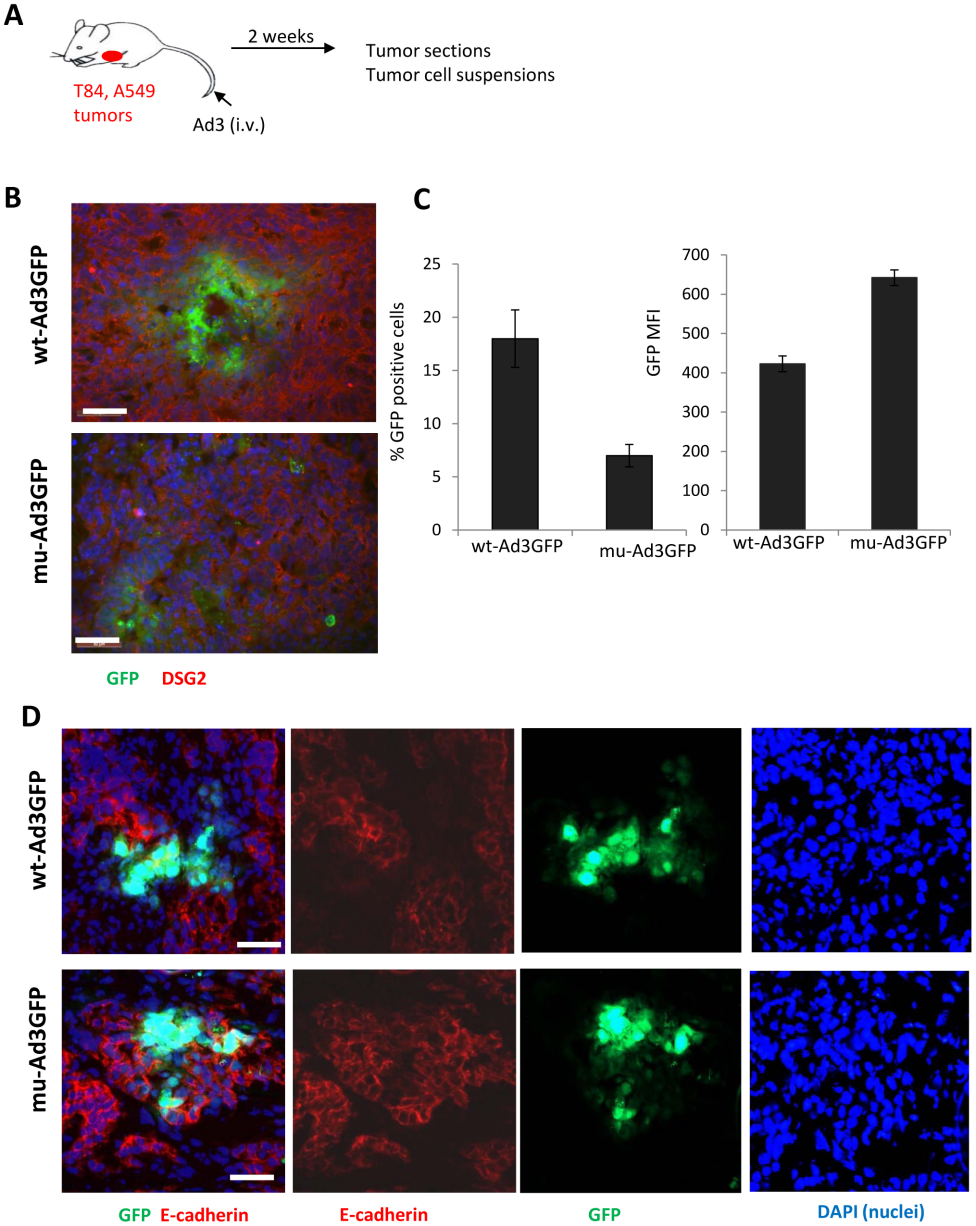
Viral spread in epithelial tumors in vivo. **A**) Scheme of experiment. Immunodeficient CB17-SCID beige mice with pre-established subcutaneous T84 or A549 tumors (∼100 mm^3^ diameter) were intravenously injected with 2×10^9^ IU of wt-Ad3GFP or mu-Ad3GFP. Fourteen days later mice were sacrificed and tumors harvested. **B**) Tumor sections were analyzed for GFP and DSG2. Representative sections are shown. The scale bar of all images is 20 µm. **C**) Tumors were digested with collagenase and dispase, and single cell suspensions were then analyzed for GFP expression by flow cytometry. N = 5. Shown is the percentage of GFP expressing cells and the mean GFP fluorescence intensity. The differences between the two viruses were significant (p<0.01). **D**) Immunofluorescence analysis of A549 tumor sections. The first (left) panels show GFP expression (green) and staining for the junction marker E-cadherin (red). The second and third panels show E-cadherin and GFP staining individually. The 4th panel shows nuclei stained with DAPI. Representative sections are shown. The staining pattern suggests that junctions around wt-Ad3GFP infected cells are absent while they are visibly present in areas of mu-Ad3GFP transduced cells. For the immunofluorescence experiments, cellular nuclei were counterstained with DAPI (blue).

T84 and A549 cells form xenograft tumors in immunodeficient mice (**Figure S11**). These tumors resemble the histology of epithelial tumors in humans. Most solid tumors are of epithelial origin and, although malignant cells are dedifferentiated, they maintain intercellular junctions both in the primary tumor as well as in metastatic lesions [11]. Unlike normal epithelial tissues, in epithelial tumors such as T84 and A549 xenografts, not all of the DSG2 molecules are trapped in epithelial junctions. DSG2-targeting Ad3 based vectors therefore efficiently transduce tumor cells after intravenous injection into mice [2], [12]. We studied viral spread of wt-Ad3GFP and mu-Ad3GFP after intravenous injection into mice with pre-established subcutaneous T84 and A549 tumors (**Figure 7A**). Upon injection, viruses were allowed to replicate in tumors for two weeks. Sections of T84 tumors from mice injected with wt-Ad3GFP showed larger foci with central necrosis and peripheral GFP expression than tumors from mu-Ad3GFP injected animals (**Figure 7B**). To quantitate the percentage of GFP expressing cells in tumors, we generated single tumor cell suspensions by protease digestion of tumors and analyzed them by flow cytometry. We found ∼3-fold more GFP-positive cells in tumors from wt-Ad3GFP injected mice compared to mu-Ad3GFP (**Figure 7C** **, upper panel**). Interestingly, the mean GFP fluorescence intensity was significantly higher in mu-Ad3GFP transduced cells (**Figure 7C** **, lower panel**). This indicates that mu-Ad3GFP is confined to the initially infected and directly adjacent cells due to the lack of PtDd production and the subsequent PtDd-mediated junction opening. In contrast, wt-Ad3GFP progeny can penetrate farther from the initial infection site due to its ability to open epithelial junctions. Similar results were obtained in the A549 xenograft model. Staining of A549 tumor sections for E-cadherin suggests that junctions around wt-Ad3GFP infected cells are absent while they are visibly present in areas of mu-Ad3GFP transduced cells (**Figure 7D**). The latter is supported by morphometry of E-cadherin signals on tumor sections. The area of E-cadherin was 6.5(+/−1.3)% and 13.3+/−1.8% (based on mm^2^ E-cadherin per mm^2^ tumor section) for wt-Ad3GFP- and mu-Ad3GFP injected mice respectively.

**Figure 8:**
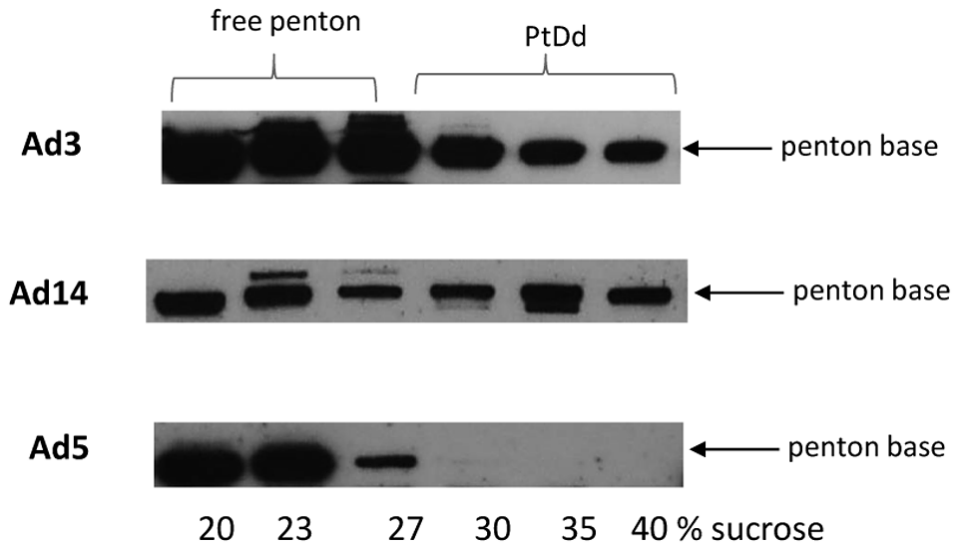
Detection of Ad14 PtDd by Western blot analysis. HeLa cells were infected with wild-type Ad3, Ad14, and Ad5 at an MOI of 500 vp/cell for 7 hours. Thirty-six hours after infection, cell lysates were subjected to ultracentrifugation on a sucrose gradient. Fractions were analyzed by Western blot using a polycloncal antibody against Ad3 PtDd, which also cross-reacts with Ad5 and Ad14 penton base.

So far, we have worked with Ad3-based vectors. The subgroup of DSG2-interacting Ad serotypes comprises Ad3, Ad7, Ad14 and Ad11. We hypothesized that these serotypes have evolved a similar strategy for viral dissemination in epithelial cells that involve the formation of PtDd. To test this, we infected HeLa cells with wild-type Ad14 virus and analyzed PtDd production in comparison to wild-type Ad3 and Ad5 virus (**Figure 8** **, Figure S12**). In this study, we capitalized on the fact that the anti-serum raised against purified Ad3 PtDd crossreacted with penton base from other serotypes. As seen before with wt-Ad3GFP, wild-type Ad3 PtDd appeared in sucrose fractions 30–40%. The signals in fractions with lower sucrose concentrations originated from free capsid proteins and defective particles. Importantly, PtDd signals were detectable for Ad14 but not for Ad5. The latter is in agreement with earlier studies demonstrating that Ad2 and Ad5 are unable to form stable pentondodecahedra [9].

## Discussion

During Ad3 infection, PtDd are produced in vast excess and released early in infection by a still unknown, non-lytic mechanism. We asked the question why Ad3 produces PtDd. In this study, we demonstrate that PtDd trigger the opening of epithelial junctions and thus support the lateral spread of Ad3 progeny virus in epithelial tissue.

In the past, most basic adenovirology studies have been done with the CAR-interacting serotypes 2 and 5. During the replication of these serotypes, penton base and fiber are also produced in excess and only a fraction is incorporated into virions. It has been reported that Ad2 penton base and fiber self-assemble into pentons. However, Ad2 pentons do not spontaneously form dodecahedra. It is thought that Ad2 pentons can form salt bridges but the penton N-termini do not rearrange as is the case for Ad3. Ad2 penton dodecamerization is only possible under artificial conditions, such as in the presence of dioxane and ammonium sulphate [9]. This raises the question why Ad3 pentons form dodecamers. The answer lies in the different mechanisms that Ad5 and Ad3 use to bind to their corresponding receptors, CAR and DSG2. High affinity Ad5 attachment involves one Ad5 fiber knob monomer and one CAR monomer, implying that one trimeric Ad5 fiber knob binds to three CAR molecules [13]. Ad5 attachment can be completely blocked by an excess of recombinant soluble fiber knob. This is not the case for Ad3. Trimeric Ad3 fiber knob was unable to block Ad3 virus binding. We showed that the efficient binding of Ad3 to DSG2 requires multiple fibers in a spatial constellation present in virions or PtDd [1]. This mode of binding triggers DSG2 clustering and subsequent intracellular signaling that results in opening of epithelial junctions [2]. Therefore for Ad3 pentons to exert a function they have to assemble into PtDd.

Furthermore, the release of Ad3 PtDd from infected cells before disruption of infected cells by *de novo* produced virions appears to be important for its function in supporting viral spread. At this time, the mechanism of PtDd release is unclear. Ad2 and Ad5 pentons are released from infected cells through a non-lytical export mechanism that does not involve Golgi vesicles or lysosomes [5]. Previous studies showed that released PtDd remain cell membrane-associated and appear to pass along inside the paracellular space [10]. Along this line, we found that after infection of HeLa cells with wt-Ad3GFP, PtDd were not efficiently released into the culture medium (**Figure S13**). This is in agreement with studies on Ad2, which failed to recover free penton base or pentons from the extracellular medium [5]. Potentially, the release of Ad3 PtDd (and Ad2 penton) involves mechanism used by proteins of other viruses to exit cells. For example, the small HIV TAT protein is released from cells by interaction of an alpha helical region and negatively charged heparan sulfate proteoglycans or phospholipids in the cell membrane [14]. It is also conceivable that Ad3 PtDd interact with DSG2 or integrins present in the endoplasmatic reticulum and that this mediates export.

As a tool to prove our hypothesis that Ad3 PtDd facilitate viral spread, we generated Ad3 penton base mutants that are disabled for the production of PtDd. Previous studies indicated that penton base mutagenesis can negatively affect protein expression and folding [15]. Based on the recently published 3D structure of Ad3 PtDd, we therefore focused on the substitution of selected amino acid residues that appeared to be critical in PtDd formation and stability. We discovered that the SELS→SDVA substitution, that would prevent the critical N-terminal strand swap, interfered with virus production. However, the double mutations (D100R and R425E) that would break up the salt bridge between two neighboring pentons had no effect on Ad3 production.

The central finding of this study is that a key function of PtDd during Ad3 virus infection is the facilitation of lateral viral spread through interaction with DSG2 and the opening of epithelial junctions. The latter is supported by our previous studies with recombinant PtDd and the recombinant dimeric Ad3 fiber knob protein JO-1 [1], [2], [3], [4], [16]. The fact that efficient junction opening can be achieved with JO-1, a protein that does not contain a penton base, argues against a role of PtDd interaction with cellular integrins in junction opening. It appears that within PtDd, the penton base has the function to bring fiber knobs into an optimal constellation for DSG2 binding and, potentially, mediate the release of PtDd from infected cells. Notably, the interaction of penton base within the complete virion with integrins is essential for virus entry into cells. As outlined above, CAR-interacting Ads have evolved another mechanism to support viral dissemination in cells where the target receptor is trapped in tight junctions and not readily accessible to Ad particles entering the body through the respiratory tract [17], [18]. A number of studies have demonstrated that during replication of Ad5, excess production of fiber or fiber/penton base complexes results in the disruption of epithelial junctions either by interfering with CAR dimerization or by triggering intracellular signaling that leads to reorganization of intercellular junctions [18], [19]. It is also noteworthy, that similar to CAR, DSG2 appears to be primarily a receptor involved in lateral spread of Ad in polarized cultures. In normal lung epithelium, DSG2 is trapped in junctions and not accessible to Ad from the apical site [2], [3]. Epithelial cancer cells (such as T84) have lost this strict polarization *in vitro* and *in vivo*, which allows for some degree of Ad3 transduction [3].

Ad3 PtDd could theoretically have a number of other functions in Ad3 infection of epithelial tissues. *i)* PtDd interaction with DSG2 and/or integrins in surrounding cells could prepare these cells for viral DNA replication, e.g. by activating the cell cycle which is particularly important in the case of non-dividing cells. *ii)* PtDd released from infected cells could mask DSG2 on infected and the neighboring cells and thus help viral dissemination to more distant cells. *iii)* As suggested by Trotman et al. [5], another function of excess production and release of PtDd could be the trapping of anti-Ad3 antibodies locally at the site of infection, but also systemically, thus avoiding the neutralization of *de novo* produced virions. PtDd could also form decoys for defensins, proteins of the immune system that suppress viral and bacterial infections. Defensins, specifically human alpha defensin 5, recognize residues/structures within the penton base and, it is thought that this interaction blocks the endosome release of adenoviral particles during infection [20]. All of the hypotheses listed above remain to be tested. Most of our studies were performed with T84 cells. This cell line forms tight junctions in vitro and has therefore been widely used in studies on the interaction of Ad5 with the tight junction protein CAR [18]. Clearly, additional studies with polarized human airway epithelia have to be conducted to consolidate our findings.

Our findings on the function of Ad3 PtDd are relevant for basic and applied virology. We provide first evidence that PtDd are also formed by another DSG2-interacting Ad serotype, Ad14. In our studies, we used a newly emerged Ad14 strain (Ad14p1) [21]. Compared to the parental strain (Ad14-deWit), Ad14p1 is more pathogenic/virulent [22], [23], [24]. Understanding how Ad spreads and penetrates through the airway epithelium also might explain how Ad3 establishes viremia and infects other tissues such as the gastro-intestinal tract. After cough and shortness of breath, the main symptoms associated with a recent Ad14p1 outbreak in the USA were vomiting and diarrhea [25]. There is a series of independent reports from different Asian, African, and South American countries stating that Ad3 infection in children is associated with acute gastroenteritis [22], [26]. We speculate that the other DSG2-targeting Ads (Ad7, Ad11) also produce PtDd. As outlined above, dodecamerization of pentons is functionally critical to trigger opening of epithelial junctions upon binding to DSG2.

The theoretical basis for cancer therapy by oncolytic adenoviruses is that a virus that specifically replicates in tumor cells spreads throughout the tumor, thus eliminating it. In reality, viral spread is blunted by anti-virus immune responses and physical barriers such as epithelial junctions, which are a hallmark of most epithelial tumors. Understanding how Ad3 as well as a number of other parasites that have evolved mechanisms to efficiently breach the epithelial barriers [27] is relevant for the development of more efficient oncolytic adenoviruses.

In summary, our study contributes to a better understanding of Ad3 infection and pathology. It also has implications for Ad-mediated gene transfer into epithelial tissues and tumors.

## Materials and Methods

### PtDd

Recombinant Ad3 penton-dodecahedra (PtDd) were produced in insect cells and purified as described previously [8].

### Antibodies

The following antibodies were used for immunofluorescence or immunoblot studies: polyclonal goat anti-DSG2 (R&D Systems, Inc, Minneapolis, MN), polyclonal goat anti-human E-Cadherin (cat. AF648, R&D Systems, Inc, Minneapolis, MN), mouse mAb anti-DSG2 (clone 6D8) (Cell Sciences, Canton, MA), anti-fiber primary antibody (Clone 4D2) (Thermo Fisher Scientific, Fremont, CA, USA), polyclonal goat anti-Ad2 (cat. 1401, Virostat, Maine, USA), monoclonal anti-actin (Sigma, St. Louis, MO), FITC conjugated goat anti-adenovirus (Millipore Billerica, MA), and PE conjugated rabbit anti-E-cadherin (BD Biosciences). The following secondary antibodies were used: anti-rabbit-AF488, anti-rabbit-AF568, anti-mouse-AF488, anti-mouse- AF568, anti-goat-AF568 (Invitrogen/Molecular Probes, Eugene, OR). Polyclonal rabbit antibodies against purified recombinant Ad3 knob were produced by PickCell Laboratories B.V. (Amsterdam, The Netherlands). The polyclonal rabbit antibody against purified recombinant Ad3 PtDd was described earlier [7]. Rhodamine labeled Concanavalin A (cat. RL-1002) and vectorshield mounting medium with DAPI (cat. H-1200) were purchased from vector laboratories (Burlingame, CA, USA).

### Cell lines

293 (Microbix, Toronto, Ontario, Canada), HeLa and A549 (American Type Culture Collection [ATCC]) cells were cultured in Dulbecco's modified essential medium (DMEM) supplemented with 10% fetal calf serum (FCS), 2 mmol/liter L-glutamine (Glu), 100 U/ml penicillin, and 100 µg/ml streptomycin (pen-strep). Colon cancer T84 cells (ATCC CCL-248) were cultured in a 1∶1 mixture of Ham's F12 medium and DMEM, 10% FBS, Glu, and pen-strep. To achieve cell polarization, 2×10^5^ T84 cells were cultured in 6.5-mm Transwell inserts (0.4 µm pore size) (Costar Transwell Clears) for more than 14 days until transepithelial resistance was stable. Culture medium was changed every 2–3 days.

#### Tumor spheroids

Exponentially growing T84 cells (3×10^7^ cells) in 150 ml fresh culture medium were transferred to a 250-ml spinner bottle (INTEGRA Biosciences, Zizers, Switzerland) and cultured in the incubator for 4 days at a stirring speed of 75 rpm. The formed spheroids were aspirated into a 15-ml tube and the medium was removed after spinning at 500 rpm for 5 min. The precipitated T84 spheroids were loosened by gentle flip and infected with viruses for 2 hours at room temperature. Unattached viruses were removed by washing, and the spheroids were resuspended in 5 ml fresh medium and cultured in the original 15-ml tube in a cell incubator. The tube was gently flipped twice every day to prevent coagulation of the spheroids. After 2–4 days' culture, spheroids were fixed in 4% paraformaldehyde (PFA) for 1 hour, washed twice with PBS, incubated with Rhodamine labeled Concanavalin A (1∶200 diluted) for 30 min at room temperature, washed another two times with PBS, transferred to a slide, mixed with vectorshield mounting medium containing DAPI, covered with a cover slip, and observed under a Zeiss LSM 510 META confocal microscope.

### Adenoviruses

Propagation and purification of Ads were performed as described elsewhere [28]. Wild-type Ad5 was rescued from pFG140 plasmid transfected 293 cells (Microbix, Toronto, Ontario, Canada). Ad14p1 (strain *Portland 2971/2007*) was provided by the Center for Disease Control and Prevention (Atlanta, GA) [21]. wt-Ad3GFP is a wild-type Ad3-based vector containing a CMV-GFP expression cassette inserted into the E3 region [2]. Penton base-mutated Ad3-GFP (mu-Ad3GFP) was constructed as following: wt-Ad3GFP plasmid (pWEA-Ad3GFP) was digested with AscI, and the short fragment (7772 bp) was ligated to an AscI-digested kanamycin resistant gene-pBR322 origin containing fragment, which was amplified from pShuttle-CMV plasmid (Stratagene, La Jolla, CA) with primers 1210KAscI01 and 1210KAscI02, to generate pKPenton3AscI (see **Table 1**). To introduce D100R mutation to pKPenton3AscI, PCR was performed with primers 1210PentonM01 and 1210PentonM02 using pKPenton3AscI as the template. PCR product (1233 bp) was excised with BamHI and NheI, and inserted into the corresponding sites of pKPenton3AscI to generate pKPenton3R. Similarly, PCR was conducted using pKPenton3AscI as the template with primers 1210PentonM03 and 04, and with primers 1210PentonM05 and 06. The two PCR products (344 and 1247 bp) were united using overlap extension PCR with primers 1210PentonM03 and 1210PentonM06. The recovered fragment (1545 bp) were digested with XbaI and XmaI, and substituted for the XbaI-XmaI region in pKPenton3R to generate pKPenton3RE. The D100R and R425E mutations in pKPenton3RE were confirmed by sequencing. pKPenton3RE was further digested with AscI, and penton-mutated fragment was inserted back to pWEA-Ad3GFP to generate a new adenovirus plasmid pWEA-mu-Ad3GFP. pWEA-mu-Ad3GFP was linearized with FseI, and then used to transfect 293 cells. The rescued viruses were propagated in HeLa cells and purified by standard methods. We have sequenced the genome of both viruses and did not find additional mutations. Whole genome sequencing was performed by Eurofins MWG Operon (Huntsville, AL).

**Table 1:** PCR primers for mu-Ad3GFP plasmid construction.

Name	sequence	Length of product (bp)	restriction enzyme site
1210KAscI01	ccggggcgcg cccgatcccg agcggtatca gctc	2492	AscI
1210KAscI02	ccggggcgcg ccaaactgga acaacactca accctatc		AscI
1210PentonM01	ccgctgagga ggagcggatc ctcagatacg	1233	BamHI
1210PentonM02	gggtgctagc ctccgtgggg gtaaacctat tgttctgcac caccgtg		NheI
1210PentonM03	cagaggaaac tagtgaagat gatgatataa c	344	SpeI
1210PentonM04	gttgttgact tgtcttgtgg actcgaaggt gactgggtct tgcatc		
1210PentonM05	gatgcaagac ccagtcacct tcgagtccac aagacaagtc aacaac	1247	
1210PentonM06	gtggtcccgg gtctgagcac ttgacgcact t		XmaI

Ad particle (viral particle [VP]) concentrations were determined spectrophotometrically by measuring the optical density at 260 nm (OD260). Infectious titers (IU/ml) of progeny viruses were determined by limiting dilution assay on 293 cells. Briefly, tenfold serially diluted virus was used to infect exponentially growing 293 cells in 96-well plates. At 48 hours postinfection, GFP-positive cells were counted under a fluorescence microscope. The infectious titer was calculated as the average number of GFP-positive cells per well×dilution factor/volume of virus suspension per well. The multiplicity of infection (MOI) was calculated from particle titer. The VP/IU ratio was 20∶1 for all virus preparations.

#### Virus growth curve

293 or T84 cells seeded in 24-well plates were infected with mu- or wt-Ad3GFP in a volume of 0.5 ml DMEM per well at indicated MOIs. Two hours later, viruses-containing media were removed. The cells were cultivated in 0.75 ml fresh DMEM plus 2% FBS per well. At indicated intervals, cells together with culture media were collected and frozen at −80°C. After all culture plates were collected, the samples were subjected to three cycles of freeze-and-thaw. The lysates were clarified by centrifugation at 12000 g for 2 minutes, and the supernatants were titrated by limiting dilution assay on 293 cells.

### Analysis of PtDd in infected HeLa cells

Hela cells in three 15-cm dishes were infected with virus (wt-Ad3GFP, mu-Ad3GFP, Ad5 or Ad14) in 20 ml medium/dish. 6–12 hours later, virus containing medium was discarded, and 15 ml fresh medium plus 2% FBS was added to each dish. 24–48 hours post infection, culture medium was aspirated to a 50-ml tube. The cells were washed once with 7 ml PBS/dish, scraped down in 4.5 ml PBS (3 dishes), and subjected to 3 rounds of freeze and thaw. Cell debris was removed by spinning at 16 100 g for 4 minutes, and the lysate supernatant was laid on the sucrose gradients. In some indicated cases, the proteins in culture medium were precipitated by addition of ammonium sulfate to a saturation of 65%, dialyzed against ultracentrifugation buffer (10 mM Tris-Cl, 150 mM NaCl, 10% glycerol, pH 7.6), and laid on sucrose gradients. After 4 hours of ultracentrifugation (SW41Ti rotor, 35,000 rpm, 200,000 g), the ultra-clear thin tube was taken out and held vertically on a clamp stand. A syringe needle (size: 18G) was used to penetrate the tube from the side above the 40% sucrose layer. Gradient fractions were collected from the needle and fractioned into tubes. The fractions were mixed with 2×SDS loading buffer, boiled for 5 minutes, loaded on 4–15% SDS-PAGE at 20 µl/well for cell-associated PtDd or 35 µl/well for released PtDd. Western blots were performed with anti-Ad3 PtDd primary antibody (dilute 1∶2000 for cell-associated PtDd or 1∶1000 for released PtDd) and anti-rabbit secondary antibody (1∶2000).

### Transduction studies

T84 cells were cultured in 0.4 µm transwell insert for 16 days, and then infected with mu-Ad3GFP or wt-Ad3GFP at MOIs of 2, 10 and 50 vp/cell for 2 hours. Supernatants were collected from the inner chambers at days 4, 7, 9 and 10 post infection, and from the outer chambers at days 9 and 10. Progeny virus in the collected culture media was titrated on 293 cells.

### Immunofluorescence staining and confocal microscopy

Cells were washed once with PBS, fixed with 4% PFA for 15 minutes at room temperature, permeabilized with 1% BSA-PBS plus 0.3% triton X-100, and hybridized with primary antibody and fluorescein labeled secondary antibody. The cellular nuclei were counterstained by mounting samples with vectorshield mounting medium containing DAPI.

Sample slides were observed under a Zeiss LSM 510 META confocal microscope. Generally, three color signals were collected with multitracking mode, and Z-stacks with a slice thickness of 1 or 2 µm were acquired.

### Image morphometry

Images of E-cadherin staining on T-84 transwell cultures and A549 tumor sections were taken using a Leica DFC300FX digital camera with a Leica DMLB microscope. Ten 20×-magnification pictures per T84 transwell (five sections per well, collected at a distance of 50 µm, 2 infected wells) and fifteen 20×-magnification pictures per A549 tumor (five sections per tumor, collected at a distance of 100 µm, 3 tumors) were analyzed using the Image-Pro Plus program (Media Cybernetics, Bethesda, MD) and the E-cadherin-positive area (% E-cadherin area) was calculated based on mm^2^ E-cadherin per mm^2^ tumor section. Technical details for morphometry have been described earlier [29].

### Permeability assay

Polarized T84 cells cultured in transwell chambers were exposed to cell lysates or gradient fractions (obtained 24 hours after infection) (20 µg total protein/ml) in adhesion medium (DMEM, 1% FBS, 2 mM MgCl_2_, 20 mM HEPES) for 15 min at room temperature and TEER was measured and calculated as described elsewhere [18].

### Animal studies

All experiments involving animals were conducted in accordance with the institutional guidelines set forth by the University of Washington. The University of Washington is an AALAC (Association for the Assessment and Accreditation of Laboratory Animal Care International) accredited research institution and all live animal work conducted at this university is in accordance with the Office of Laboratory Animal Welfare (OLAW) Public Health Assurance (PHS) policy, USDA Animal Welfare Act and Regulations, the Guide for the Care and Use of Laboratory Animals and the University of Washington's Institutional Animal Care and Use Committee (IACUC) policies. The studies were approved by the University of Washington IACUC (protocol # 3108-01). Mice were housed in specific-pathogen-free facilities. T84 cell and A549 xenograft tumors were established by subcutaneous injection of 2×10^6^ cells. Ad3 virus (2×10^9^ IU/mouse) was intravenously injected. Tumors were harvested 10 days later. Half of the tumor was used for cryosection. The other half was digested with collagenase/displays to generate single cell suspensions as described earlier [30]. GFP expression in tumor cell suspensions was analyzed by flow cytometry.

### 3D model of penton base dodecahedron

Ad3 penton base structure (PDB 4AQQ) was imaged using Pymol software. Polar contact between monomers of two adjacent penton bases was calculated by Pymol software. In the green overall Bs-Dd structure, the salt bridges were highlighted by drawing D100 and R425 in red and blue spheres respectively.

### Statistical analysis

All results are expressed as mean +/− SD. 2-Way ANOVA for multiple testing was applied. Animal numbers and P values are indicated in the figure legends.

## Supporting Information

Figure S1 **Western blot analysis of PtDd produced in insect and human cells.** The filter was hybridized with polyclonal rabbit anti-PtDd and anti-rabbit HRP antibodies. Lanes 1 and 2: 100 and 50 ng of purified recombinant PtDd produced from baculovirus vectors in insect cells. Lanes 3, 4, 5: PtDd purified from a lysate of HeLa infected for 24 hours by wt-Ad. Loading corresponds to about 1×10^6^; 5×10^5^ and 2.5×10^5^ infected HeLa cells, respectively.
(TIF)

Figure S2 **Detection of D100R and R425E point mutations in the genome of mu-Ad3GFP by PCR.** DNA was isolated from purified viral particles and used as PCR template. Primers were designed and synthesized as following: for D100R, ggcaacccgt tcgctcatct and gcctccgtgg gggtaaacct; for R425E, agaggaaac tagtgaagat gatgatataa c and gtagttgttg acttgtcttg tggactc; for D100, ggcaacccgt tcgctcatct and gcctccgtgg gggtaaagtc; and for R425, agaggaaac tagtgaagat gatgatataa c and tgttgacttg tcttgtggag cg. Taq DNA polymerase was used. There is a three-nucleotide difference at the 3′ end for mutation site binding primers used to amplify mutated and wild-type sequence. Therefore, positive band could be seen with mu-Ad3GFP template using D100R primers (for mutant detection), while no band could be amplified with mu-Ad3GFP template using D100 primers (for wild type detection), and vice versa. The result was further confirmed by sequencing.
(TIF)

Figure S3 **Semiquantitative analysis of mature virions by measuring genomic DNA content.** Virus that pelleted at the bottom of tube after ultracentrifugation of culture medium concentrate or cell lysates in sucrose gradients was collected, viral DNA was isolated and analyzed by electrophoresis in 0.5% agarose gels. No mature virions were detected in culture medium at 36 hours post infection, while the yields of mature progeny virus of mu-Ad3GFP and wt-Ad3GFP inside cells were comparable. M. lambda/HindIII fragments.
(TIF)

Figure S4 **Detection of PtDd and defective viral particles by Western blot.** **A and B**) HeLa cells were infected with wt-Ad3GFP or mu-Ad3GFP at an MOI of 1800 vp/cell for 12 hours. Cells were harvested at either 24 hours (**A**) or 48 hours (**B**). Cell lysates were subjected to ultracentrifugation and fractions were analyzed by Western blot using polyclonal antibodies raised against purified PtDd (upper panels) or anti-adenovirus antibody (lower panels). V1,V2: purified wt-Ad3GFP 8.3×10^8^, 2.5×10^9^ vp. **C**) Evaluation of relative yield of PtDd in wt-Ad3GFP and mu-Ad3GFP-infected HeLa cells 48 hours post-infection. Hexon, penton and fiber bands were visualized in Western blot by using anti-adenovirus antibody. We assume that hexon band represented the slight contamination of empty virion during PtDd purification, the contamination of empty virion was similar in intensity between mu-Ad3GFP and wt-Ad3GFP, and the density of penton base represented the amount of PtDd. Namely, hexon band could be used to normalize PtDd signals. Based on quantitative image analysis of Western blot signal, it could be calculated that the amount of PtDd in wt-Ad3GFP-infected Hela cells was about 10 times of that in mu- Ad3GFP-infected cells after normalized with hexon band. mu-Ad3GFP, 30% sucrose fraction; wt-Ad3GFP, 30% sucrose fraction; EV, empty/defective Ad3GFP virus (the upper band from the first round of Ad3GFP purification on CsCl gradients); PV, purified wt-Ad3GFP; rPtDd, purified recombinant PtDd expressed in insect cells.
(TIF)

Figure S5 **Analysis of PtDd containing fractions by Western blot.** Western blot was performed as described in Figure S4. Hela cells were infected with mu-Ad3GFP or wt-Ad3GFP at an MOI of 500 vp/cell. 48 hours-post infection, the cells were harvested in PBS and subjected to 3 rounds of freeze and thaw. Cell debris was removed by spinning and the supernatant was laid on the sucrose gradient (15–45%). After ultracentrifugation, sucrose fractions were collected and loaded on 4–15% SDS-PAGE. Western blot were performed with anti-Ad5 fiber antibodies (upper panel) and anti-Ad3 PtDd antibodies (lower panel). rPtDd, purified recombinant PtDd expressed in insect cells. The amount of loaded purified viruses was 4×10^9^ vp.
(TIF)

Figure S6 **Analysis of transepithelial electrical resistance (TEER) measured on polarized T84 cells.** **A**) T84 cells were cultured in transwell chambers until the TEER was constant, i.e. tight junctions had formed. A total of 10 µl from the 30% sucrose fraction collected from wt-Ad3GFP and mu-Ad3GFP infected cells was added for 1 hour to the apical chamber. TEER was measured at the indicated time points. Control is 30% sucrose only. The TEER (average) at 1.5 hours was 4759, 2315, and 4120 Ω/cm2 for the control, wt-Ad3GFP, and mu-Ad3GFP material, respectively. For time points 1.5 and 4 hours the difference between wt-Ad3GFP and mu-Ad3GFP was significant (p<0.01). N = 6. **B**) T84 cells were exposed to 10 µl from the 30% sucrose fractions mixed with 30 µg/ml of recombinant DSG2 as described above. TEER was measured at 1.5 hours. N = 3
(TIF)

Figure S7 **Confocal microscopy of wt-Ad3GFP infected HeLa cells.** HeLa cells were infected for 24 hours with wt-Ad3GFP and stained anti-PtDd antibodies followed by anti-rabbit Cy3. Nuclei were labelled with DAPI. The arrows show GFP-positive, infected cells. The stars show non-infected but PtDd positive cells.
(TIF)

Figure S8 **Tight junctions formed in T84 spheroids.** T84 spheroids were analyzed by confocal immunofluorescence microscopy with antibodies against DSG2 (upper panel) or E-cadherin and claudin 7 (lower panel). The lower panel is a selection of XY slices through two spheroids.
(TIF)

Figure S9 **Confocal microscopy for GFP in T84 cell spheroids.** T84 spheroids were infected with mu-Ad3GFP or wt-Ad3GFP at an MOI of 100 vp/cell for 2 hours. Viruses were removed by washing. Spheroids were fixed with 4% PFA at day 4 post-infection. The periphery of spheroids was visualized with rhodamine-labeled Concanavalin A (Con A, red), and cellular nuclei were counterstained with DAPI (blue). Transduced cells are GFP positive. Shown are serial sections of the spheroids. The scale bars are 25 µm.
(TIF)

Figure S10 **Viral spread in monolayers of A549 cells.** **A**) Titration of Ad produced in A549 cells. Cells were infected at indicated MOIs (vp/cell) for 2 hours. Medium samples were collected at indicated time points. Viruses were titrated on 293 cells based on GFP-expressing units (“Infectious Units”). N = 3. **B**) Release of PtDd from mu-Ad3GFP or wt-Ad3GFP-infected A549 cells. A549 cells were infected with mu-Ad3GFP or wt-Ad3GFP at an MOI of 10 vp/cell for 2 hours. 24 hours post infection, cells were fixed in 4% PFA for 15 minutes and then permeabilized with cold methanol for 10 minutes. Immunofluorescence staining was performed with anti-Ad3 PtDd polyclonal antibody (red). GFP expression results in green signals. **C and D**) A549 cells in 6-well plate were infected with mu-Ad3GFP or wt-Ad3GFP at an MOI of 0.01 vp/cell. Two hours post infection, viruses were removed and semisolid medium (DMEM containing 0.5% agarose and 2% FBS) was added to infected cultures. 10 days post infection, GFP foci were counted under a fluorescence microscope according to their size (**C**). The largest GFP focui formed in mu-Ad3GFP-infected and in wt-Ad3GFP-infected wells were photographed at day 9 post infection *(* **D** *)*.
(TIF)

Figure S11 **Immunofluorescence analysis of sections from tumors derived from T84 or A549 cells.** Human epithelial tumor cells were injected subcutaneously into CB17-SCID/beige mice. When tumors reached a volume of 200 mm^3^, sections were stained with antibodies against DSG2 and claudin 7. The scale bars are 20 µm.
(TIF)

Figure S12 **Kinetics of Ad14 PtDd formation.** HeLa cells were infected with wt-Ad14 at an MOI of 500 vp/cell for 7 hours. Cells were collected in PBS at 25, 33, 48 hours post-infection and lysates were subjected to ultracentrifugation in sucrose gradients. Fractions corresponding to 23% and 35% sucrose were analyzed by Western blot with anti-Ad3 PtDd antibody. PV, purified Ad3GFP virus (1×10^9^ vp); Ad14, Ad14 virus pellet collected from the bottom of ultracentrifugation tube (48 h).
(TIF)

Figure S13 **Release of PtDd from in wt-Ad3GFP-infected HeLa cells into the culture supernatant.** HeLa cells were infected with wt-Ad3GFP at an MOI of 1000 vp/cell. Culture media (supernatant) and cells were collected at 24, 36 and 48 hours post-infection, respectively. Proteins in the supernatant were precipitated by addition of ammonium sulfate to a saturation of 65%, and then dissolved in and further dialyzed again in ultracentrifugation buffer. The concentrated proteins in the supernatant and lysate of the cells were applied on 15%–40% sucrose gradient, and subjected to ultracentrifugation. 30%–40% fractions (PtDd) were collected, loaded in SDS-PAGE and analyzed with Western blot using anti-penton antibody. M, protein markers; PV, purified wt-Ad3GFP of 8.3×10^8^ vp.
(TIF)
