# Supplementary figures and images for: Penton-Dodecahedral Particles Trigger Opening of Intercellular Junctions and Facilitate Viral Spread during Adenovirus Serotype 3 Infection of Epithelial Cells
Source: PLoS Pathog. 2013 Oct 31;9(10):e1003718. doi: 10.1371/journal.ppat.1003718 (PMC3814681; doi:10.1371/journal.ppat.1003718)

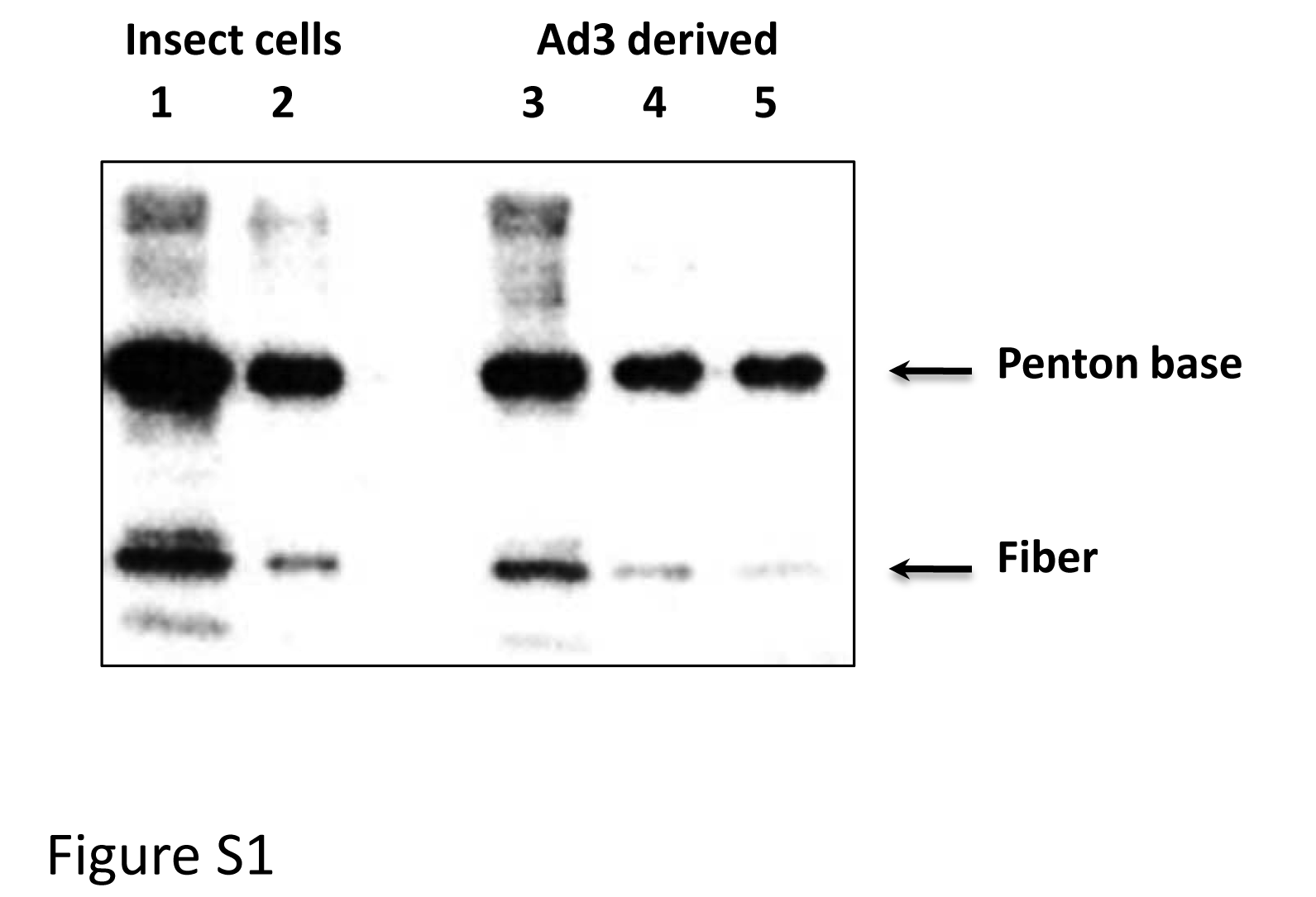

Supplement: Figure S1 — Western blot analysis of PtDd produced in insect and human cells. The filter was hybridized with polyclonal rabbit anti-PtDd and anti-rabbit HRP antibodies. Lanes 1 and 2: 100 and 50 ng of purified recombinant PtDd produced from baculovirus vectors in insect cells. Lanes 3, 4, 5: PtDd purified from a lysate of HeLa infected for 24 hours by wt-Ad. Loading corresponds to about 1×10^6^; 5×10^5^ and 2.5×10^5^ infected HeLa cells, respectively. (TIF) [file ppat.1003718.s001.tif]

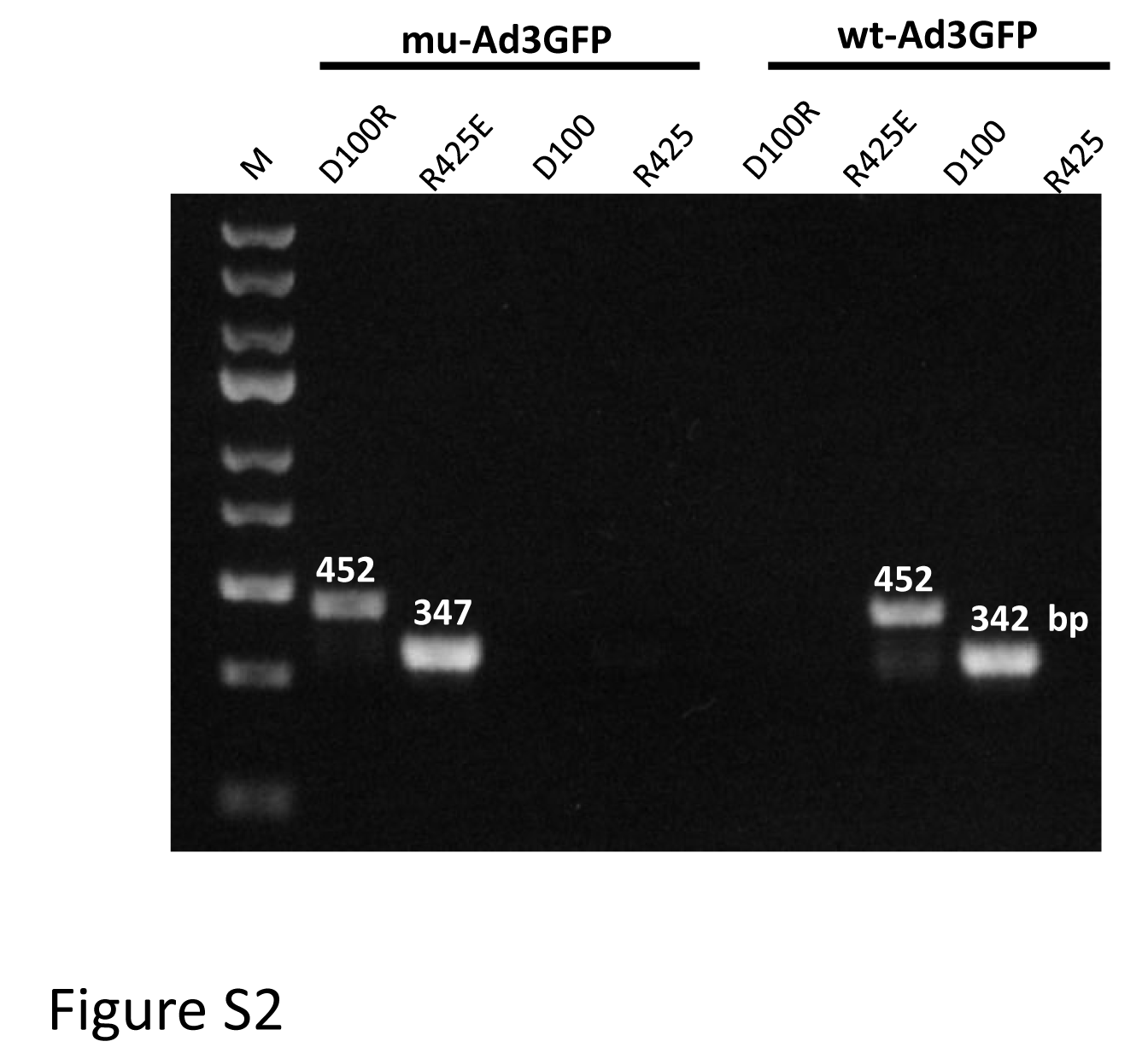

Supplement: Figure S2 — Detection of D100R and R425E point mutations in the genome of mu-Ad3GFP by PCR. DNA was isolated from purified viral particles and used as PCR template. Primers were designed and synthesized as following: for D100R, ggcaacccgt tcgctcatct and gcctccgtgg gggtaaacct; for R425E, agaggaaac tagtgaagat gatgatataa c and gtagttgttg acttgtcttg tggactc; for D100, ggcaacccgt tcgctcatct and gcctccgtgg gggtaaagtc; and for R425, agaggaaac tagtgaagat gatgatataa c and tgttgacttg tcttgtggag cg. Taq DNA polymerase was used. There is a three-nucleotide difference at the 3′ end for mutation site binding primers used to amplify mutated and wild-type sequence. Therefore, positive band could be seen with mu-Ad3GFP template using D100R primers (for mutant detection), while no band could be amplified with mu-Ad3GFP template using D100 primers (for wild type detection), and vice versa. The result was further confirmed by sequencing. (TIF) [file ppat.1003718.s002.tif]

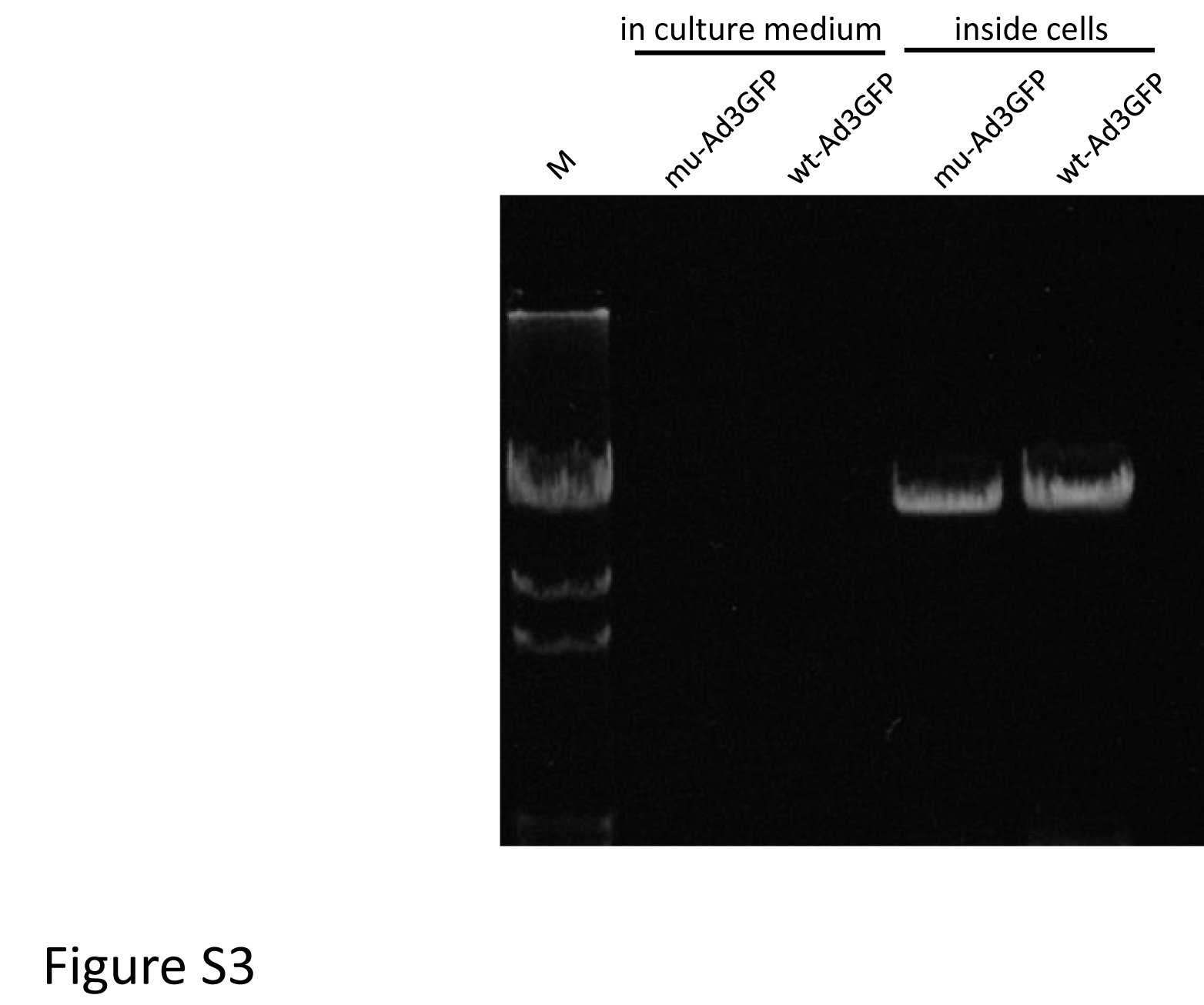

Supplement: Figure S3 — Semiquantitative analysis of mature virions by measuring genomic DNA content. Virus that pelleted at the bottom of tube after ultracentrifugation of culture medium concentrate or cell lysates in sucrose gradients was collected, viral DNA was isolated and analyzed by electrophoresis in 0.5% agarose gels. No mature virions were detected in culture medium at 36 hours post infection, while the yields of mature progeny virus of mu-Ad3GFP and wt-Ad3GFP inside cells were comparable. M. lambda/HindIII fragments. (TIF) [file ppat.1003718.s003.tif]

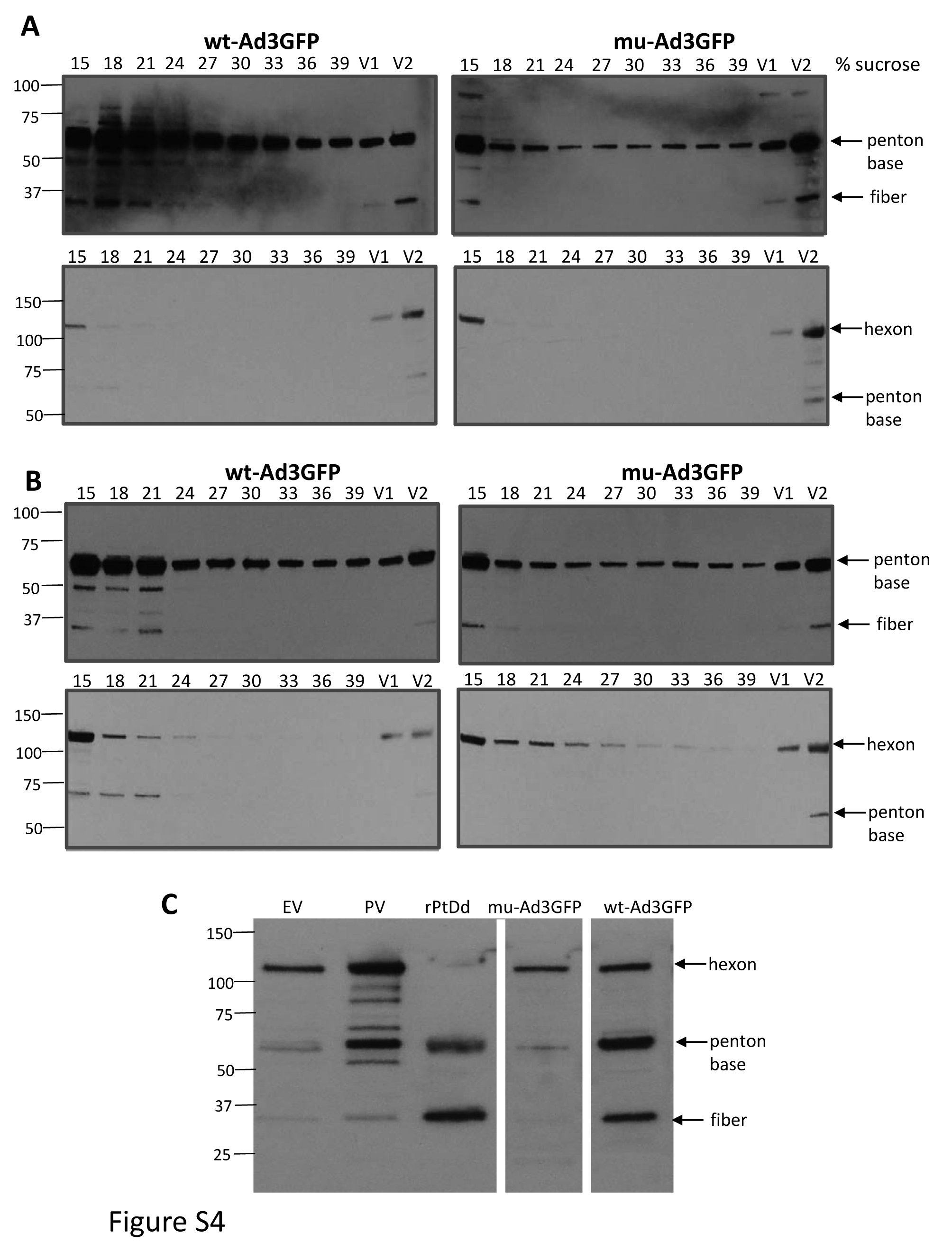

Supplement: Figure S4 — Detection of PtDd and defective viral particles by Western blot. A and B) HeLa cells were infected with wt-Ad3GFP or mu-Ad3GFP at an MOI of 1800 vp/cell for 12 hours. Cells were harvested at either 24 hours (A) or 48 hours (B). Cell lysates were subjected to ultracentrifugation and fractions were analyzed by Western blot using polyclonal antibodies raised against purified PtDd (upper panels) or anti-adenovirus antibody (lower panels). V1,V2: purified wt-Ad3GFP 8.3×10^8^, 2.5×10^9^ vp. C) Evaluation of relative yield of PtDd in wt-Ad3GFP and mu-Ad3GFP-infected HeLa cells 48 hours post-infection. Hexon, penton and fiber bands were visualized in Western blot by using anti-adenovirus antibody. We assume that hexon band represented the slight contamination of empty virion during PtDd purification, the contamination of empty virion was similar in intensity between mu-Ad3GFP and wt-Ad3GFP, and the density of penton base represented the amount of PtDd. Namely, hexon band could be used to normalize PtDd signals. Based on quantitative image analysis of Western blot signal, it could be calculated that the amount of PtDd in wt-Ad3GFP-infected Hela cells was about 10 times of that in mu- Ad3GFP-infected cells after normalized with hexon band. mu-Ad3GFP, 30% sucrose fraction; wt-Ad3GFP, 30% sucrose fraction; EV, empty/defective Ad3GFP virus (the upper band from the first round of Ad3GFP purification on CsCl gradients); PV, purified wt-Ad3GFP; rPtDd, purified recombinant PtDd expressed in insect cells. (TIF) [file ppat.1003718.s004.tif]

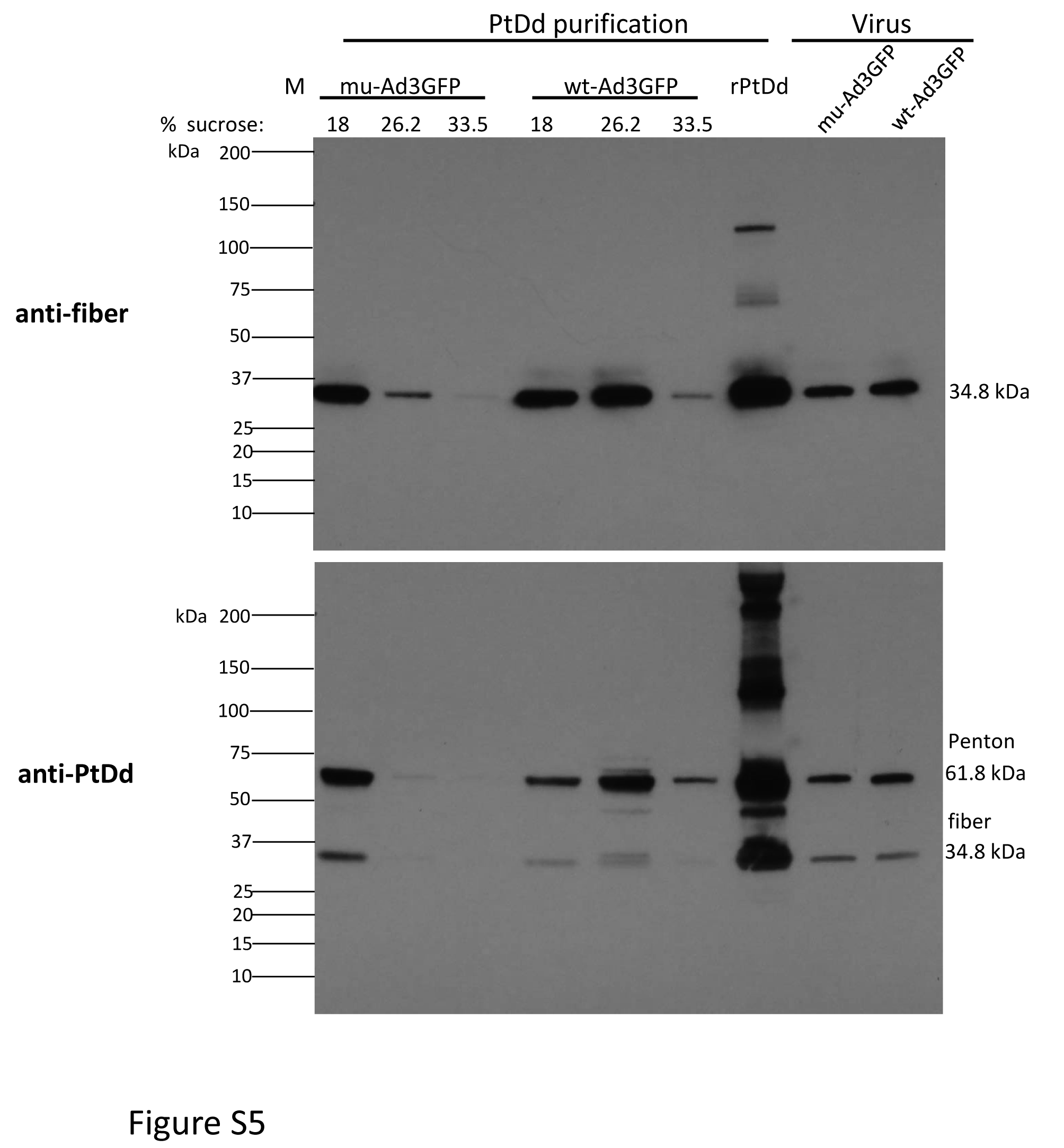

Supplement: Figure S5 — Analysis of PtDd containing fractions by Western blot. Western blot was performed as described in Figure S4. Hela cells were infected with mu-Ad3GFP or wt-Ad3GFP at an MOI of 500 vp/cell. 48 hours-post infection, the cells were harvested in PBS and subjected to 3 rounds of freeze and thaw. Cell debris was removed by spinning and the supernatant was laid on the sucrose gradient (15–45%). After ultracentrifugation, sucrose fractions were collected and loaded on 4–15% SDS-PAGE. Western blot were performed with anti-Ad5 fiber antibodies (upper panel) and anti-Ad3 PtDd antibodies (lower panel). rPtDd, purified recombinant PtDd expressed in insect cells. The amount of loaded purified viruses was 4×10^9^ vp. (TIF) [file ppat.1003718.s005.tif]

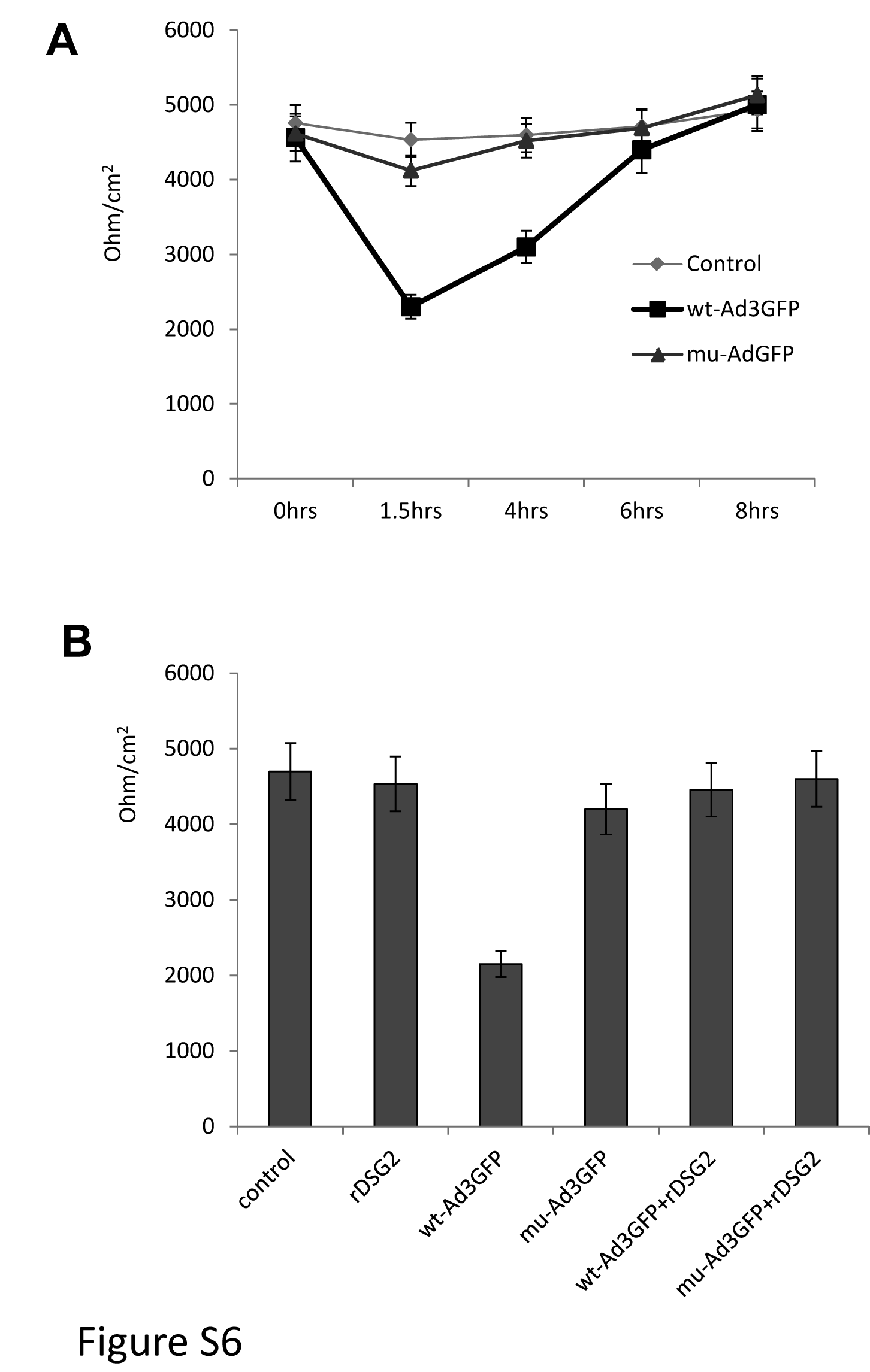

Supplement: Figure S6 — Analysis of transepithelial electrical resistance (TEER) measured on polarized T84 cells. A) T84 cells were cultured in transwell chambers until the TEER was constant, i.e. tight junctions had formed. A total of 10 µl from the 30% sucrose fraction collected from wt-Ad3GFP and mu-Ad3GFP infected cells was added for 1 hour to the apical chamber. TEER was measured at the indicated time points. Control is 30% sucrose only. The TEER (average) at 1.5 hours was 4759, 2315, and 4120 Ω/cm2 for the control, wt-Ad3GFP, and mu-Ad3GFP material, respectively. For time points 1.5 and 4 hours the difference between wt-Ad3GFP and mu-Ad3GFP was significant (p<0.01). N = 6. B) T84 cells were exposed to 10 µl from the 30% sucrose fractions mixed with 30 µg/ml of recombinant DSG2 as described above. TEER was measured at 1.5 hours. N = 3 (TIF) [file ppat.1003718.s006.tif]

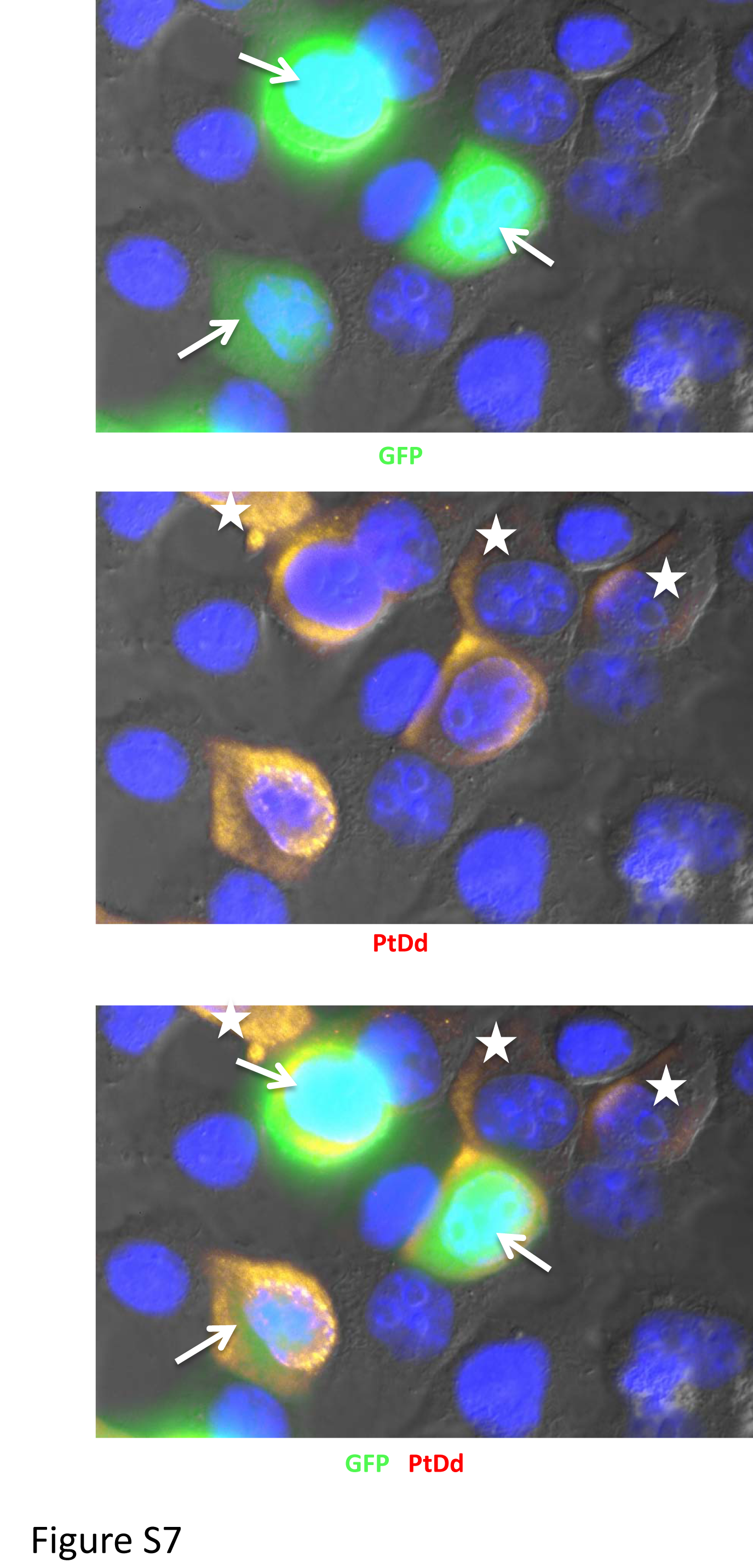

Supplement: Figure S7 — Confocal microscopy of wt-Ad3GFP infected HeLa cells. HeLa cells were infected for 24 hours with wt-Ad3GFP and stained anti-PtDd antibodies followed by anti-rabbit Cy3. Nuclei were labelled with DAPI. The arrows show GFP-positive, infected cells. The stars show non-infected but PtDd positive cells. (TIF) [file ppat.1003718.s007.tif]

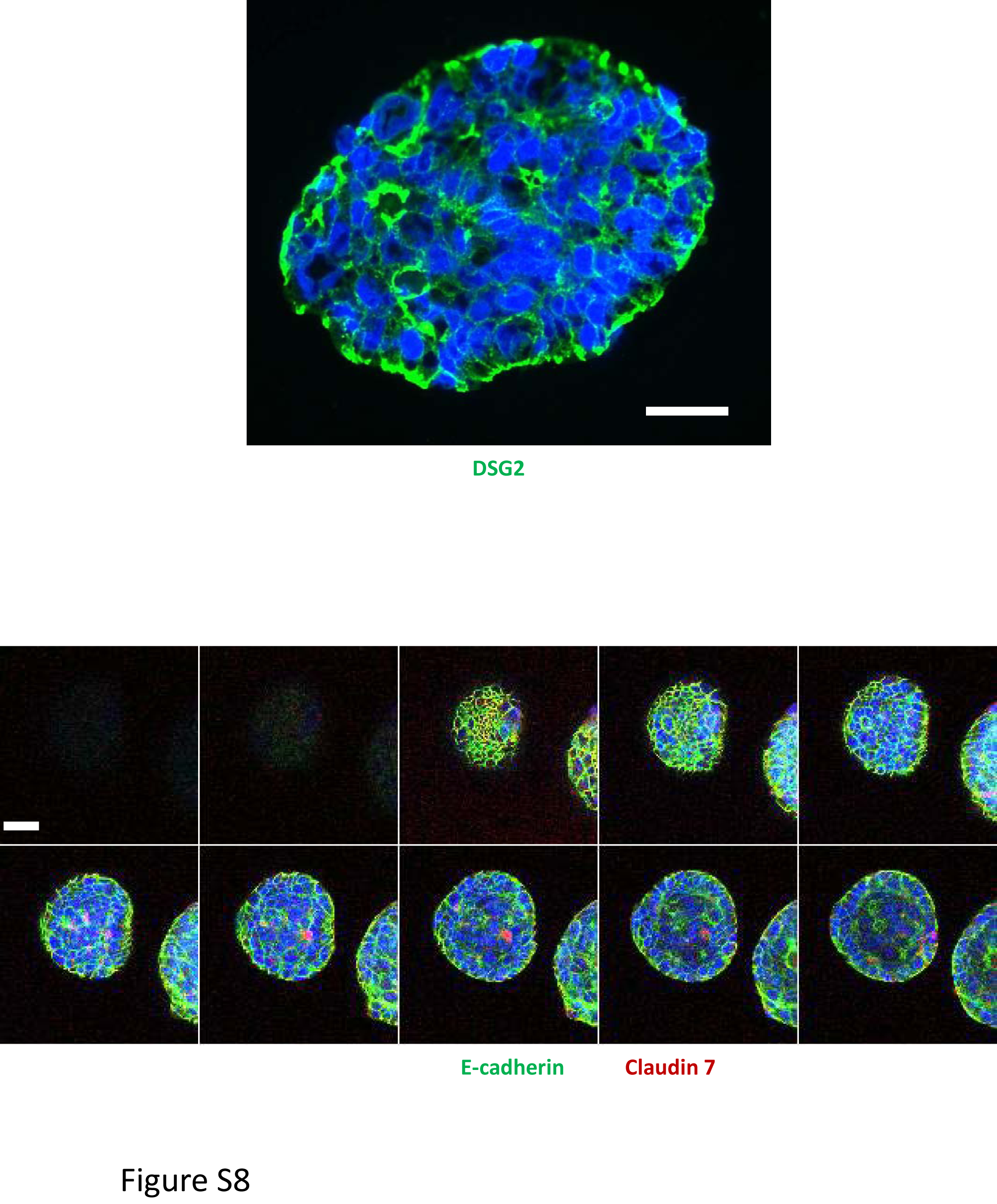

Supplement: Figure S8 — Tight junctions formed in T84 spheroids. T84 spheroids were analyzed by confocal immunofluorescence microscopy with antibodies against DSG2 (upper panel) or E-cadherin and claudin 7 (lower panel). The lower panel is a selection of XY slices through two spheroids. (TIF) [file ppat.1003718.s008.tif]

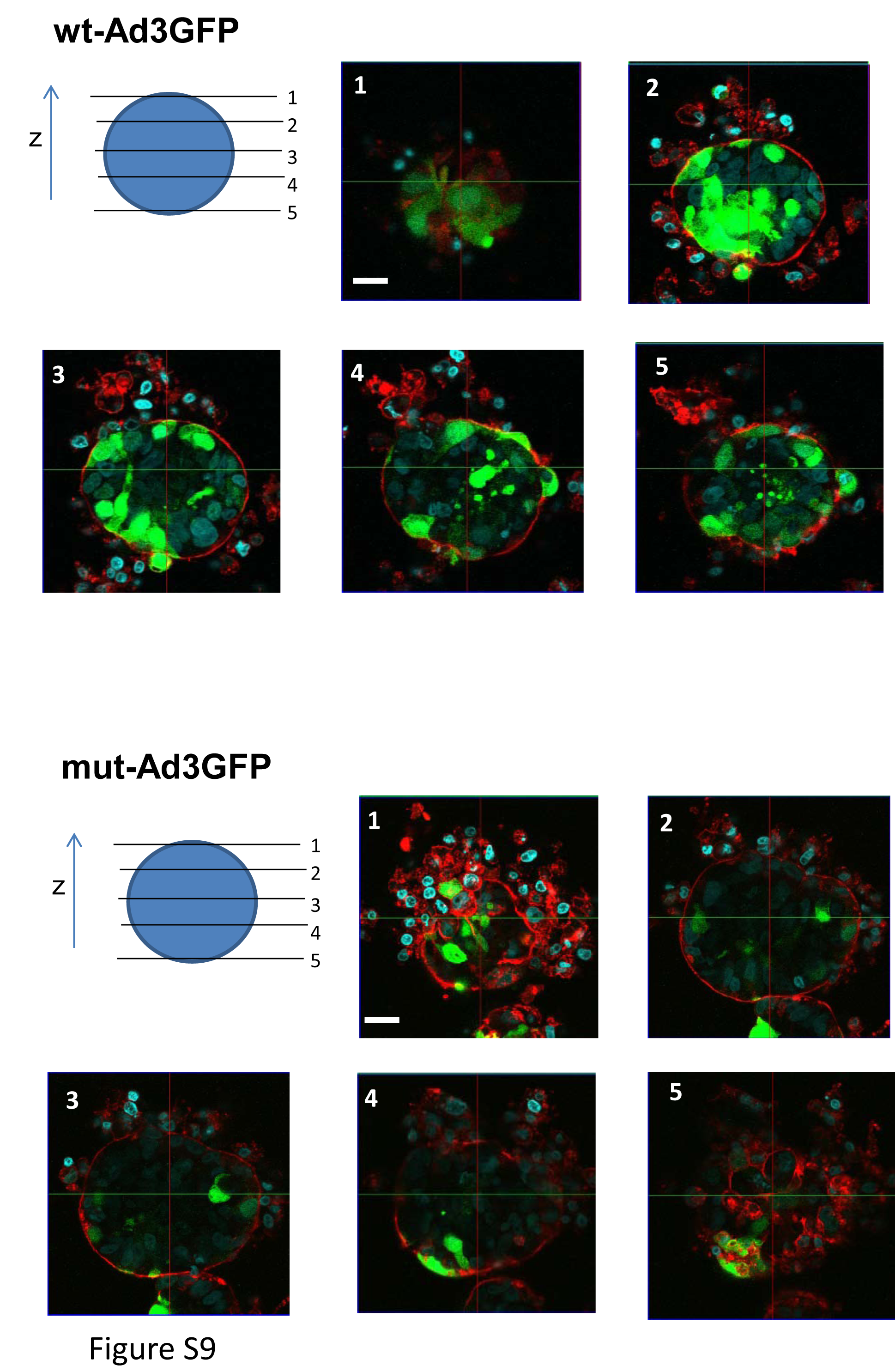

Supplement: Figure S9 — Confocal microscopy for GFP in T84 cell spheroids. T84 spheroids were infected with mu-Ad3GFP or wt-Ad3GFP at an MOI of 100 vp/cell for 2 hours. Viruses were removed by washing. Spheroids were fixed with 4% PFA at day 4 post-infection. The periphery of spheroids was visualized with rhodamine-labeled Concanavalin A (Con A, red), and cellular nuclei were counterstained with DAPI (blue). Transduced cells are GFP positive. Shown are serial sections of the spheroids. The scale bars are 25 µm. (TIF) [file ppat.1003718.s009.tif]

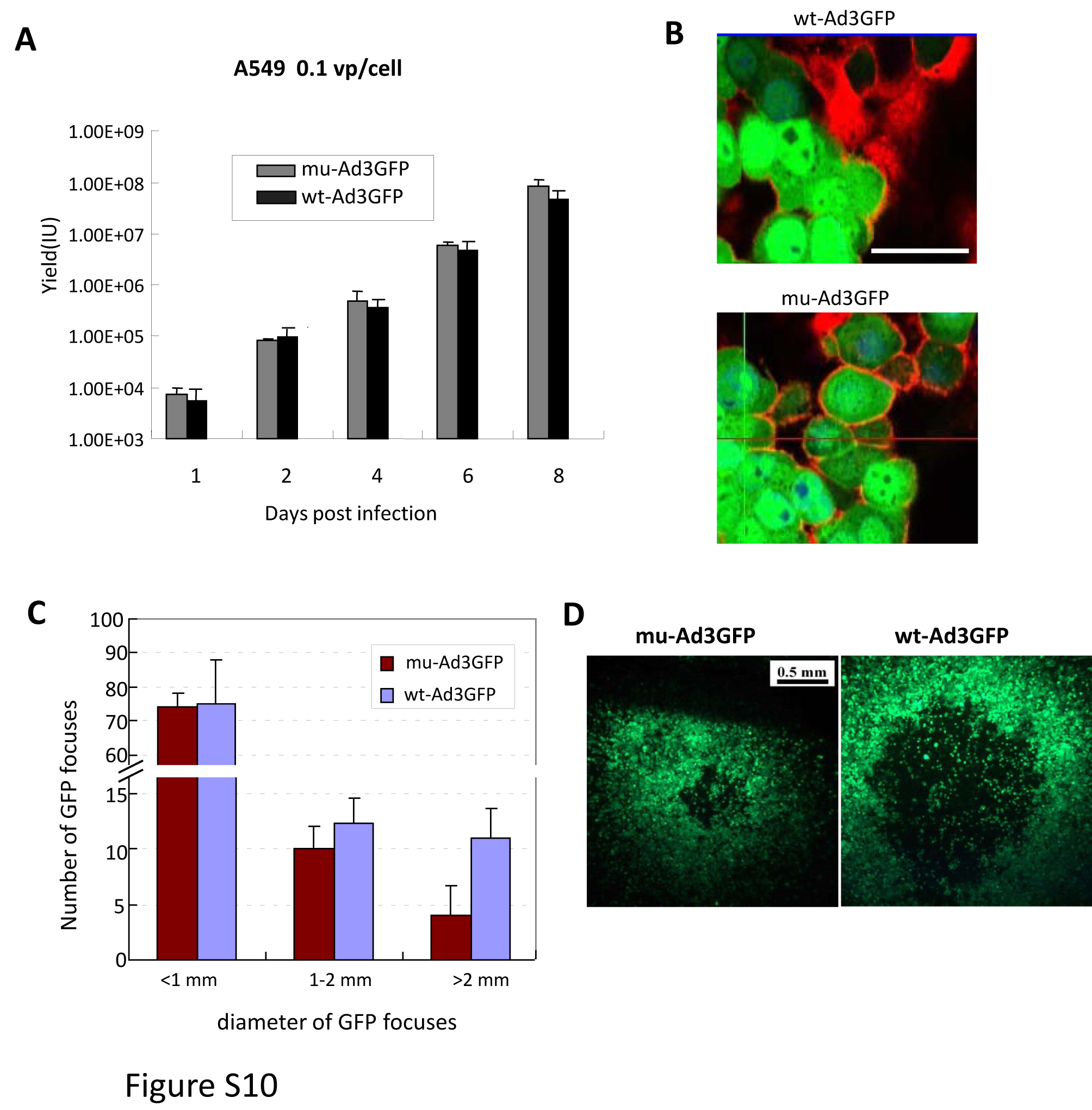

Supplement: Figure S10 — Viral spread in monolayers of A549 cells. A) Titration of Ad produced in A549 cells. Cells were infected at indicated MOIs (vp/cell) for 2 hours. Medium samples were collected at indicated time points. Viruses were titrated on 293 cells based on GFP-expressing units (“Infectious Units”). N = 3. B) Release of PtDd from mu-Ad3GFP or wt-Ad3GFP-infected A549 cells. A549 cells were infected with mu-Ad3GFP or wt-Ad3GFP at an MOI of 10 vp/cell for 2 hours. 24 hours post infection, cells were fixed in 4% PFA for 15 minutes and then permeabilized with cold methanol for 10 minutes. Immunofluorescence staining was performed with anti-Ad3 PtDd polyclonal antibody (red). GFP expression results in green signals. C and D) A549 cells in 6-well plate were infected with mu-Ad3GFP or wt-Ad3GFP at an MOI of 0.01 vp/cell. Two hours post infection, viruses were removed and semisolid medium (DMEM containing 0.5% agarose and 2% FBS) was added to infected cultures. 10 days post infection, GFP foci were counted under a fluorescence microscope according to their size (C). The largest GFP focui formed in mu-Ad3GFP-infected and in wt-Ad3GFP-infected wells were photographed at day 9 post infection ( D ). (TIF) [file ppat.1003718.s010.tif]

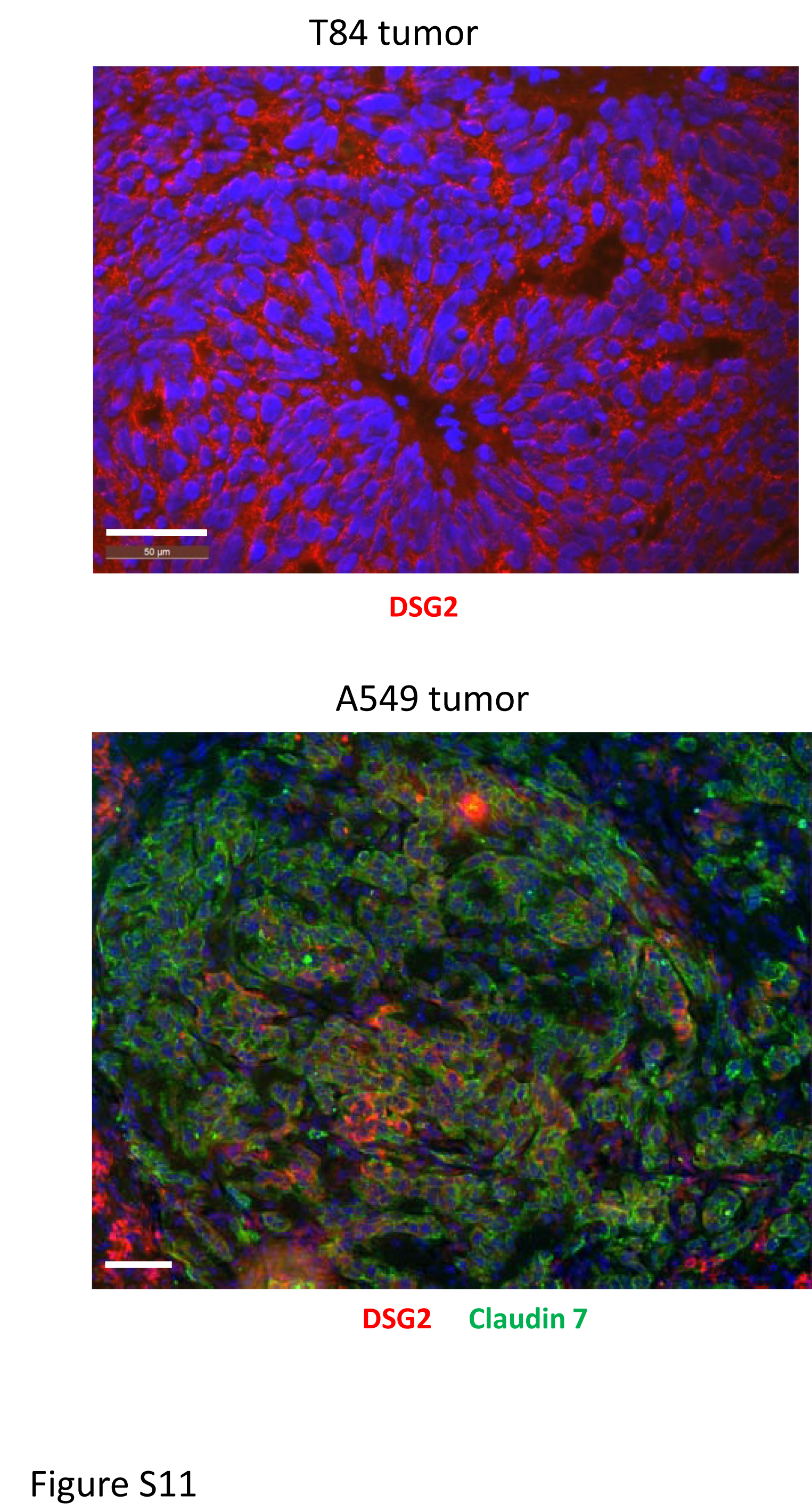

Supplement: Figure S11 — Immunofluorescence analysis of sections from tumors derived from T84 or A549 cells. Human epithelial tumor cells were injected subcutaneously into CB17-SCID/beige mice. When tumors reached a volume of 200 mm3, sections were stained with antibodies against DSG2 and claudin 7. The scale bars are 20 µm. (TIF) [file ppat.1003718.s011.tif]

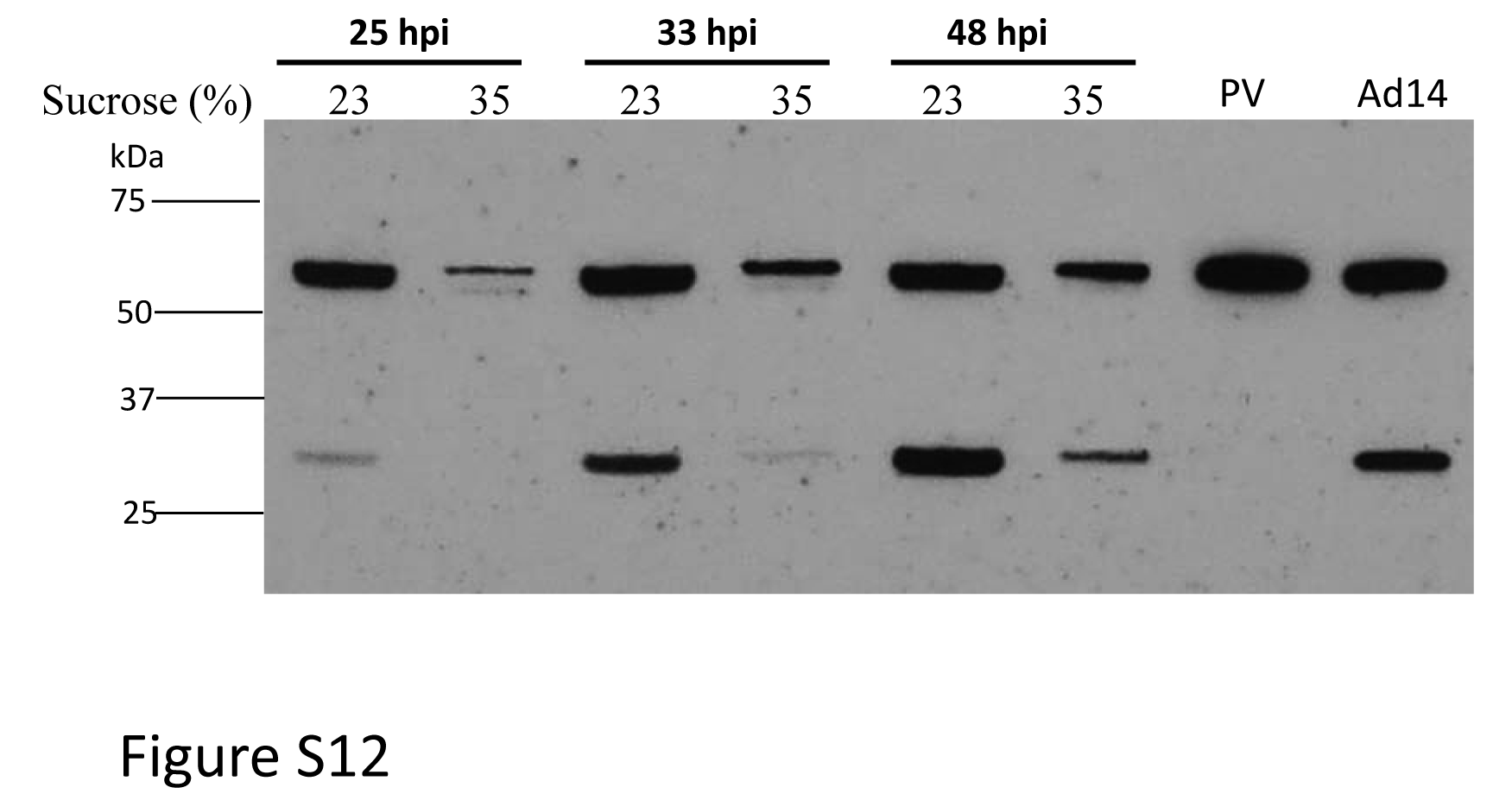

Supplement: Figure S12 — Kinetics of Ad14 PtDd formation. HeLa cells were infected with wt-Ad14 at an MOI of 500 vp/cell for 7 hours. Cells were collected in PBS at 25, 33, 48 hours post-infection and lysates were subjected to ultracentrifugation in sucrose gradients. Fractions corresponding to 23% and 35% sucrose were analyzed by Western blot with anti-Ad3 PtDd antibody. PV, purified Ad3GFP virus (1×10^9^ vp); Ad14, Ad14 virus pellet collected from the bottom of ultracentrifugation tube (48 h). (TIF) [file ppat.1003718.s012.tif]

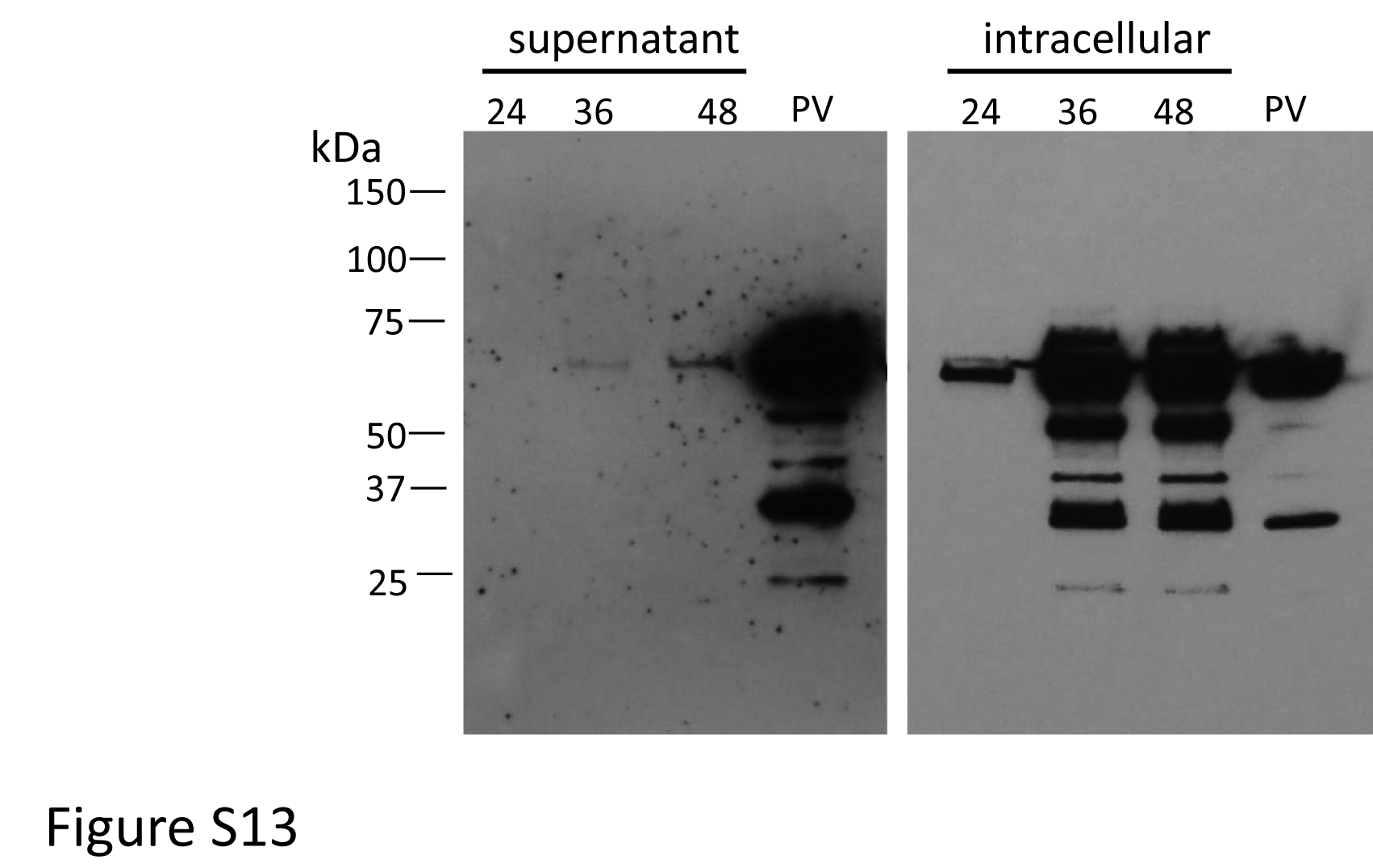

Supplement: Figure S13 — Release of PtDd from in wt-Ad3GFP-infected HeLa cells into the culture supernatant. HeLa cells were infected with wt-Ad3GFP at an MOI of 1000 vp/cell. Culture media (supernatant) and cells were collected at 24, 36 and 48 hours post-infection, respectively. Proteins in the supernatant were precipitated by addition of ammonium sulfate to a saturation of 65%, and then dissolved in and further dialyzed again in ultracentrifugation buffer. The concentrated proteins in the supernatant and lysate of the cells were applied on 15%–40% sucrose gradient, and subjected to ultracentrifugation. 30%–40% fractions (PtDd) were collected, loaded in SDS-PAGE and analyzed with Western blot using anti-penton antibody. M, protein markers; PV, purified wt-Ad3GFP of 8.3×10^8^ vp. (TIF) [file ppat.1003718.s013.tif]
